# Plasma FGF2 and YAP1 as novel biomarkers for MCI in the elderly: analysis via bioinformatics and clinical study

**DOI:** 10.3389/fnins.2025.1663276

**Published:** 2025-08-26

**Authors:** Yejing Zhao, Xiang Wang, Jie Zhang, Yanyan Zhao, Yi Li, Ji Shen, Ying Yuan, Jing Li

**Affiliations:** ^1^Department of Cardiology, Beijing Anzhen Hospital, Capital Medical University, Beijing, China; ^2^Department of Geriatrics, Institute of Geriatric Medicine, Beijing Hospital, National Center of Gerontology, Chinese Academy of Medical Sciences, Beijing, China; ^3^Department of Cardiology, Institute of Geriatric Medicine, Beijing Hospital, National Center of Gerontology, Chinese Academy of Medical Sciences, Beijing, China

**Keywords:** Alzheimer’s disease, mild cognitive impairment, FGF2, Yap1, biomarkers

## Abstract

The contemporary consensus firmly emphasizes the urgent need to reorient research efforts toward the early detection of preclinical Alzheimer’s disease (AD) or mild cognitive impairment (MCI). However, there is still a notable absence of novel biomarkers that are both efficient, minimally invasive, and cost-effective in real-world clinical settings. To address this gap, datasets GSE29378 and GSE12685 were selected to screen differentially expressed genes (DEGs), and hub genes were identified by different algorithms. A total of 350 DEGs were identified in bioinformatics data mining. Functional enrichment analysis showed that fibroblast growth factor 2(FGF2) and yes-associated protein 1(YAP1) protein levels were highly expressed in AD samples, indicating their potential regulatory roles in AD. Between October and November 2024, a total of 146 elderly individuals diagnosed with MCI and 54 healthy elderly subjects were successfully recruited. Enzyme linked immunosorbent assay (ELISA) was used to detect plasma hub gene protein concentration. The results showed that the expression levels of plasma FGF2 and YAP1 proteins in the MCI group were significantly higher compared to the control group. Logistic regression analysis indicated that high plasma FGF2 and YAP1 expression levels were independently associated with MCI in the elderly. The Area under the curve (AUC) of FGF2 model and YAP1 model were 0.907 and 0.972, respectively. Therefore, the high expression of plasma FGF2 and YAP1 proteins may be independent predictive risk factors for MCI in the elderly. Our findings may provide targets for the development of early minimally invasive, efficient, and convenient screening tools, and even for the treatment of AD in the future.

## 1 Introduction

As the problem of population aging deteriorates, Alzheimer’s disease (AD) has become one of the main causes of disability in the elderly (Gustavsson et al., 2023). As of 2023, more than 55 million people worldwide are estimated to suffer from AD, and this number is expected to increase to 82 million by 2030 and 150 million by 2050 (GBDS Collaborators, 2022). Elderly people may suffer from the progressive loss of intellectual and social abilities that will eventually interfere with daily functioning, that is costly not only as a terminal disease but also economically and socially. According to statistical reports, the annual socio-economic cost of AD in China was 168 billion US dollars in 2015, and it is expected to increase to 1890 billion US dollars by 2050 (Jia et al., 2018). The clinical spectrum of AD encompasses a range from asymptomatic stages to mild cognitive impairment (MCI) and, eventually, to mild, moderate, or severe AD. Once it progresses to the stage of dementia, the process of AD will accelerate and cannot be reversed (Vermunt et al., 2019). Therefore, there is a pressing need to shift the focus toward the early detection of preclinical AD or MCI. Early diagnosis and treatment of MCI can play an important role in delaying the onset of AD, improving the quality of life of patients, and alleviating the burden on families and society. However, MCI is characterized by cognitive decline that does not significantly impair daily life activities. The early identification and diagnosis of MCI remain challenging at present, due to its heterogeneity and concealment of clinical presentation (Albert et al., 2011). As auxiliary tools for detecting and diagnosing the disease, novel biomarkers have become a field of great interest for both clinicians and researchers. Development of new biomarkers may shift the diagnosis of AD from the later dementia stages of disease toward the earlier stages and introduce the potential for pre-symptomatic diagnosis.

The abnormal accumulation of amyloid beta (Aβ) and tau protein in the brain measured by positron emission tomography (PET), as well as the levels of Aβ42 and phosphorylated tau181 (p-tau181) in cerebrospinal fluid (CSF), are the current core biomarkers of AD (Rabinovici et al., 2019; Hansson, 2021). However, the high cost, low throughput, and radiation exposure of PET and the invasiveness of lumbar puncture highlight the necessity and urgency of exploring alternative markers in the blood. In recent years, some blood biomarkers have been studied extensively, such as Aβ Tau protein, p-tau181Aβ Tau protein, and glial fibrillary acidic protein (GFAP), which may be used to predict the progression of AD (Palmqvist et al., 2020; Hansson et al., 2023; Rissman et al., 2024). However, there is still some controversy over the current conclusions. At the same time, due to the significantly lower concentration in the blood compared to CSF, the requirements for detection technology and costs are higher, which also hinders the development and promotion of these biomarkers in clinical practice. Therefore, there is an urgent need to explore novel biomarkers with high diagnostic efficacy, minimally invasive, and acceptable detection costs for better early identification of MCI in clinical practice.

In the study, two AD datasets GSE29378 and GSE12685 profiles were downloaded from the Gene Expression Omnibus (GEO), followed by screening and enrichment of differentially expressed genes (DEGs). Finally, a bioinformatic analysis of DEGs relevant to AD was conducted to identify hub genes. On this basis, we further conducted the clinical research to recruit the elderly with MCI, and explored whether the plasma expression levels of these genes could serve as new biomarkers for MCI in the elderly, providing new insights for early identification and diagnosis of cognitive impairment.

## 2 Materials and methods

### 2.1 Bioinformatics mining of hub genes

#### 2.1.1 Datasets

GEO is a gene expression database created and maintained by the National Center for Biotechnology Information. It contains high-throughput gene expression data submitted by research institutions worldwide. We obtained the AD datasets GSE29378 and GSE12685 profiles from GEO. The GSE29378 dataset contained 31 AD samples and 32 control samples; GSE12685 contained 6 AD samples and 8 control samples. These datasets were used to identify DEGs in AD.

#### 2.1.2 Repeatability test for the datasets

Principal Component Analysis (PCA) was used to test the repeatability of the GSE29378 and GSE12685 datasets. The R programming language is an operating environment for statistical analysis and graph plotting, and was used to visualize the distribution and differences between AD and control samples.

#### 2.1.3 Screening of differently expressed genes

The Linear Models for Microarray Data (‘Limma’) R package was used for probe summarization and background correction of the GSE29378 and GSE12685 matrices. The Benjamini-Hochberg method was used to adjust the raw *p*-values. Fold changes (FC) were calculated using the false discovery rate (FDR). The cut-off value for DEGs was *p* < 0.05, and FC ≥ 1.5. A volcano map was drawn using the volcano plotting tool to identify DEGs.

#### 2.1.4 Weighted gene co-expression network analysis

WGCNA is a method of summarizing gene expression data into co-expression modules. We used the Good Samples Genes method of R package WGCNA to remove abnormal genes and samples, and constructed a scale-free co-expression network. The Pearson’s correlation matrix and average linkage method were applied to all paired genes, and then a weighted adjacency matrix was constructed using a power function (a_mn_ = | c_mn_ |*^β^*). β was a soft threshold power, that was mainly related to the independence and average connectivity degree in co-expression modules. The topological overlap measure (TOM) represented the overlap of network neighbors, and 1-TOM retrieved a pairwise distance to identify hierarchical clustering nodes and modules. Clustering graphs were performed by application of dynamic tree cutting technique in R software.

#### 2.1.5 Construction and analysis of protein protein interaction networks

The Search Tool for the Retrieval of Interacting Genes (STRING) database^1^ is an online search database for known protein interactions and was used for the construction of PPI network. The list of differential genes was input into the STRING database to construct a PPI network for predicting hub genes (confidence>0.4). Cytoscape software was used to visualize and predict hub genes from the PPI network.

#### 2.1.6 Functional enrichment analysis

Gene Ontology (GO) analysis annotates the functions of genes using terms from a dynamic, controlled vocabulary based on three aspects of biology: biological processes (BP), cellular components (CC) and molecular functions (MF). Kyoto Encyclopedia of Genes and Genomes (KEGG)^2^ contains information about specific pathways and links genomic information with higher-order functional information. Gene set enrichment analysis (GSEA)^3^ is a computational method that conducts GO and KEGG analyses for a given gene list, and is used to analyze genome-wide expression profiling data on a chip. Additionally, Metascape integrates many authoritative data resources, such as GO, KEGG, UniProt and DrugBank, to perform pathway enrichment, biological process annotation, gene related protein network analysis and drug analysis. In our study, GO and KEGG analyses of DEGs were performed using GSEA, and Metascape (*p* < 0.05).

#### 2.1.7 Comparative toxicogenomics database analysis

The CTD integrates extensive data on interactions between chemicals, genes, functional phenotypes, and diseases, facilitating research on disease-related environmental exposure factors and potential drug mechanisms. The hub genes were inputted into the CTD website to identify diseases most closely related to these hub genes and created radar charts in Excel to display expression differences for each gene.

#### 2.1.8 Gene expression heatmap

The R package heatmap was used to create heatmaps of the expression levels of hub genes identified by three algorithms in the PPI network across GSE29378 and GSE12685. The visual heatmap highlighted the expression differences of hub genes between the disease group and the control group.

### 2.2 Clinical trial validation of potential target genes

#### 2.2.1 Study design and participants

A total of 200 older adults aged ≥ 60 living in the community in Beijing were recruited between October, 2024 to November, 2024. Participants were excluded if they had impaired consciousness, tremor, parkinsonism, higher brain dysfunction such as aphasia or apraxia, epilepsy, paralysis, sensory disturbance, presence of severe liver or kidney disease, severe hematologic disorders or malignant tumors, presence of other severe illnesses with a life expectancy < 1 year or diagnosed with dementia.

The Beijing Hospital, National Center of Gerontology, Institute of Geriatric Medicine, Chinese Academy of Medical Sciences approved the study (Approval No. 2024BJYYEC-KY200-02). Written informed consents were obtained from all participants.

#### 2.2.2 Cognitive function assessment and diagnostic criteria

The cognitive function of all participants was assessed by the Montreal Cognitive Assessment (MoCA). Participants were further divided into the MCI group and the control group based on the Peterson criteria (Petersen, 2004).

#### 2.2.3 Clinical data

The trained physicians collected basic information of participants through face-to-face interviews, including age, sex, marital status, job type and status, residential status, educational level, household income and past medical history. Body weight and height were measured, and body mass index (BMI, kg/m^2^) was calculated. Functional status was evaluated by measuring calf circumference, grip strength, and Short Physical Performance Battery (SPPB).

#### 2.2.4 Detection of plasma hub gene proteins

Fasting venous blood samples were collected from all participants in the morning. Plasma was obtained using heparin as an anticoagulant, followed by centrifugation at 3000 rpm for 30 min. Plasma levels of fibroblast growth factor 2 (FGF2) and yes-associated protein 1(YAP1) were measured using commercial Enzyme linked immunosorbent assay (ELISA) kits: Human FGF2 ELISA Kit (Cat. No. MM-13497H1, MEIMIAN, Yancheng, China; sensitivity <1.0 pg./mL, detection range 1.0–400 pg./mL) and Human YAP1 ELISA Kit (Cat. No. MM-1779H1, MEIMIAN, Yancheng, China; sensitivity <10 pg./mL, detection range 10–1,200 pg./mL). All assays were performed according to the manufacturer’s instructions, with specific steps detailed below.

ELISA plates were removed from 4 °C storage and equilibrated at room temperature for 20 min before use. The required strips were taken out, and the remaining ones were sealed and returned to 4°C. For the standard wells, 50 μL of standards at varying concentrations were added. For the sample wells, 10 μL of plasma and 40 μL of sample diluent were mixed thoroughly. No reagents were added to the blank wells. Next, 100 μL of horseradish peroxidase conjugated detection antibody was added to each standard and sample well (excluding blank wells). The plate was sealed and incubated at 37°C for 60 min. After incubation, the liquid was discarded, and the plate was gently tapped dry on absorbent paper. Each well was then washed with buffer, left to stand for 1 min, and tapped dry; this washing step was repeated five times. Then, 50 μL of substrate solution A and 50 μL of substrate solution B were added to each well, followed by incubation at 37°C in the dark for 15 min. Finally, 50 μL of stop solution was added to each well, and the optical density (OD) at 450 nm was measured within 15 min using a microplate reader (SpectraMax iD3, Molecular Devices, San Jose, United States).

### 2.3 Statistical analysis

All statistical analyses were conducted using SPSS software (version 26.0; IBM Corp, Armonk, NY, United States). The results of categorical variables were expressed as *n* or percentage, and continuous variables were expressed as mean ± standard deviation (SD) or median quartile. Student’s *t* test was used for normally distributed variables, and Mann–Whitney *U* test was used for asymmetrically distributed variables. The comparison between categorical variables used χ^2^ test. The multiple logistic regression was used to determine the relationship between plasma hub gene proteins concentration and cognitive function. Receiver Operating Characteristic (ROC) curve analysis was used to evaluate diagnostic efficacy. Area under the curve (AUC) and its 95% confidence interval (CI) were also calculated for each model, and *p* value < 0.05 was considered significant.

## 3 Results

### 3.1 Hub genes FGF2 and YAP1 are highly expressed in AD by bioinformatics

**Figure 1:**
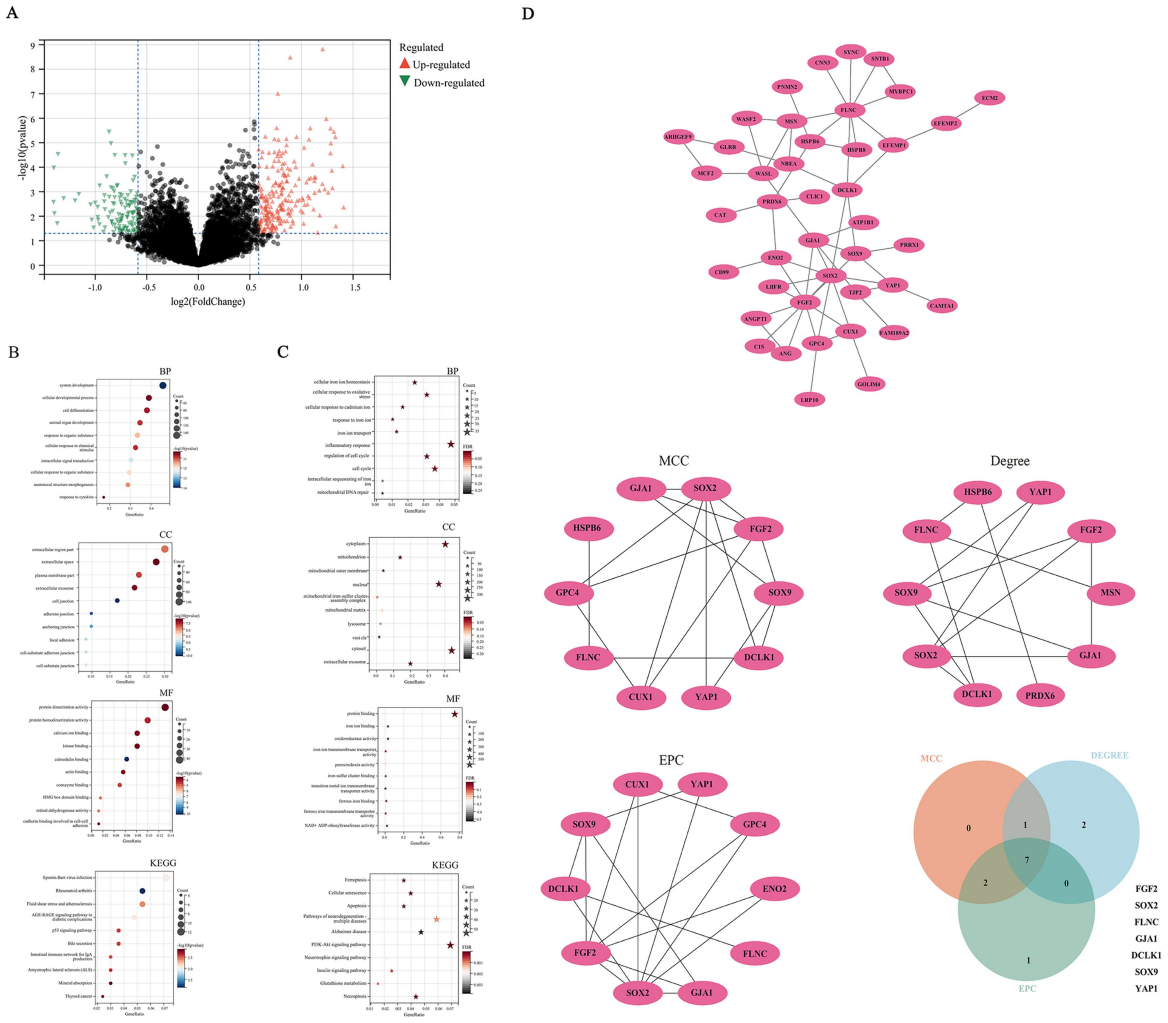
Bioinformatics mining of hub genes. **(A)** Differential gene analysis. **(B)** function enrichment analysis of DEGs; **(C)** function enrichment analysis on genes associated with ferroptosis; **(D)** construction and analysis of protein–protein interaction networks.

PCA was used to reduce dimensionality and visualize these data samples, revealing the underlying structure of the data and discovering biological features of differences (Supplementary Figure 1). In bioinformatics data mining and analysis, 350 DEGs were identified from the GSE29378 and GSE12685 matrices (Figure 1A). GO and KEGG analyses were conducted on these DEGs shown in Figure 1B. In addition, we conducted GSEA enrichment analysis on the entire genome to validate the results of DEGs, and performed Metascape enrichment analysis to provide a comprehensive gene list annotation and resource analysis for DEGs (Supplementary Figure 2).

With the extensive researches on regulatory cell death in AD, more and more evidence has revealed that ferroptosis is closely related to the occurrence, development, and outcome of AD (Kwiatek-Majkusiak et al., 2015; Hambright et al., 2017; Ryu et al., 2019; Yan and Zhang, 2019). In view of this, the present study conducted enrichment functional analysis on genes associated with ferroptosis furtherly. Functional enrichment analysis revealed DEGs were primarily enriched in cellular iron homeostasis, oxidative stress response, inflammatory response, as well as ferroptosis, neurodegenerative pathways, and AD (Figure 1C; Supplementary Figure 3). We further conduct WGCNA analysis and PPI network. Seven hub genes, namely FGF2, SRY-Box transcription factor 2(SOX2), filamin C(FLNC), gap junction protein alpha 1(GJA1), doublecortin like kinase 1(DCLK1), SRY-Box transcription Factor 9(SOX9), YAP1,were obtained by taking the intersection using a Venn diagram (Figure 1D; Supplementary Figures 4, 5). CTD analysis suggested that the seven hub genes were associated with immune system diseases, brain injury, and deposition of beta amyloid plaques (Supplementary Figure 6). A heatmap was constructed to visualize the expression profiles of hub genes. The FDR for the respective molecules were as follows: YAP1 with an FDR of 0.003, FGF2 with an FDR of 0.017, SOX2 with an FDR of 0.217, FLNC with an FDR of 0.184, GJA1 with an FDR of 0.085, DCLK1 with an FDR of 0.085, and SOX9 with an FDR of 0.264. FGF2 and YAP1 proteins exhibited statistically significant differences, characterized by upregulation in AD samples and downregulation in normal controls. This observation indicates their potential regulatory roles in AD pathogenesis, thereby prompting their selection for subsequent in-depth investigation and validation (Supplementary Figure 7).

### 3.2 Demographic characteristics of participants in clinical trial

A total of 200 participants were recruited, of which 146 participants experienced MCI. Table 1 showed a comparison of demographic characteristics of the participants between the MCI group and the control group. There were no statistically significant differences in all variables including age, gender, education level, medical history, physical function, etc., except for a slight difference in BMI between the two groups.

**Table 1:** Demographic characteristics and cognitive function assessment of the participants.

Demographics	All	Control group	MCI group	*p*
	*n* = 200	*n* = 54	*n* = 146	
Age (years)	67.51(5.62)	66.96(6.86)	67.71(5.10)	0.408
Female (%)	84(42.00)	25(46.30)	59(40.40)	0.454
Marital status (%)				0.194
Unmarried/Divorced/Widowed	13(6.50)	1(1.85)	12(8.22)	
Married	187(93.50)	53(98.14)	134(91.78)	
Occupation type (%)				0.082
Mental work	121(60.50)	38(70.37)	83(56.85)	
Manual work	79(39.50)	16(29.63)	63(43.15)	
Residency status (%)				0.351
Live alone	11(5.50)	1(1.85)	10(6.85)	
Live with spouse	149(74.50)	42(77.78)	107(73.29)	
Live with children	40(20.00)	11(20.37)	29(19.86)	
Education (%)				0.630
Junior school and below	64(32.00)	15(27.78)	49(33.56)	
Senior high school/Technical secondary school/Junior college	112(56.00)	31(57.41)	81(55.48)	
Undergraduate degree and above	24(12.00)	8(14.81)	16(10.96)	
Household income (%)				0.779
<5,000	27(13.50)	6(11.11)	21(14.38)	
5,000–10,000	80(40.00)	25(46.30)	55(37.67)	
10,000–30,000	89(44.50)	22(40.74)	67(45.89)	
>30,000	4(2.00)	1(1.85)	3(2.05)	
BMI (Kg/m^2^)	24.65(3.23)	23.90(2.43)	24.92(3.44)	0.048
Calf circumference (cm)	35.58(3.90)	35.04(4.14)	35.79(3.80)	0.227
Past medical history (%)				
Hypertension	94(47.00)	20(37.04)	74(50.68)	0.110
Hyperlipidemia	40(20.00)	10(18.52)	30(20.55)	0.844
Thyroid Disease	3(1.50)	1(1.85)	2(1.37)	1.000
Diabetes	39(19.50)	9(16.67)	30(20.55)	0.558
CVD	14(7.00)	1(1.85)	13(8.90)	0.118
Smoking history (%)				0.974
Never	127(63.50)	35(64.81)	92(63.01)	
Quit smoking	35(17.50)	9(16.67)	26(17.81)	
Smoking	38(19.00)	10(18.52)	28(19.18)	
Drinking history (%)				0.423
Never	154(77.00)	39(72.22)	115(78.77)	
Quit drinking	11(5.50)	5(9.26)	6(4.11)	
Drinking	35(17.50)	10(18.52)	25(17.12)	
Gait speed (m/s)	3.56(0.78)	3.67(0.70)	3.52(0.81)	0.241
Five-Times-Sit-to-Stand Test (s)	3.62(0.79)	3.61(0.83)	3.62(0.77)	0.923
SPPB score	11.13(1.37)	11.20(1.38)	11.10(1.38)	0.628
Grip (Kg)	29.61(8.79)	29.31(8.74)	29.72(8.83)	0.768
Global Cognitive Function				
MoCA score	23.29(2.97)	27.04(0.87)	21.90(2.17)	<0.001
Cognitive Functional Dimensions				
Visuospatial Executive	3.95(0.96)	4.59(0.09)	3.71(0.07)	<0.001
Naming	2.63(0.67)	2.85(0.06)	2.54(0.06)	0.010
Attention	5.44(0.78)	5.85(0.04)	5.28(0.06)	<0.001
Language	1.94(0.75)	2.37(0.08)	1.78(0.61)	<0.001
Abstraction	1.41(0.72)	1.75(0.07)	1.28(0.60)	<0.001
Delayed Recall	2.02(1.63)	3.66(0.13)	1.41(0.11)	<0.001
Orientation	5.89(0.36)	5.94(0.03)	5.86(0.03)	0.077

MCI, Mild cognitive impairment; BMI, Body mass index; CVD, Cardiovascular disease; SPPB, Short physical performance battery; MoCA, Montreal Cognitive Assessment.

### 3.3 Cognitive function of participants in clinical trial

The MoCA scale was used to evaluate the cognitive function of participants, as shown in Table 1. The average score for the MCI group was 21.90 ± 2.17 points, while the control group was 27.04 ± 0.87 points, indicating a significant decline in global cognitive function among the elderly with MCI. Except for the orientation dimension, all dimensions of cognitive function showed significant decline in the MCI group compared to the control group,

### 3.4 Comparison of plasma FGF2 and YAP1 protein expression levels

**Figure 2:**
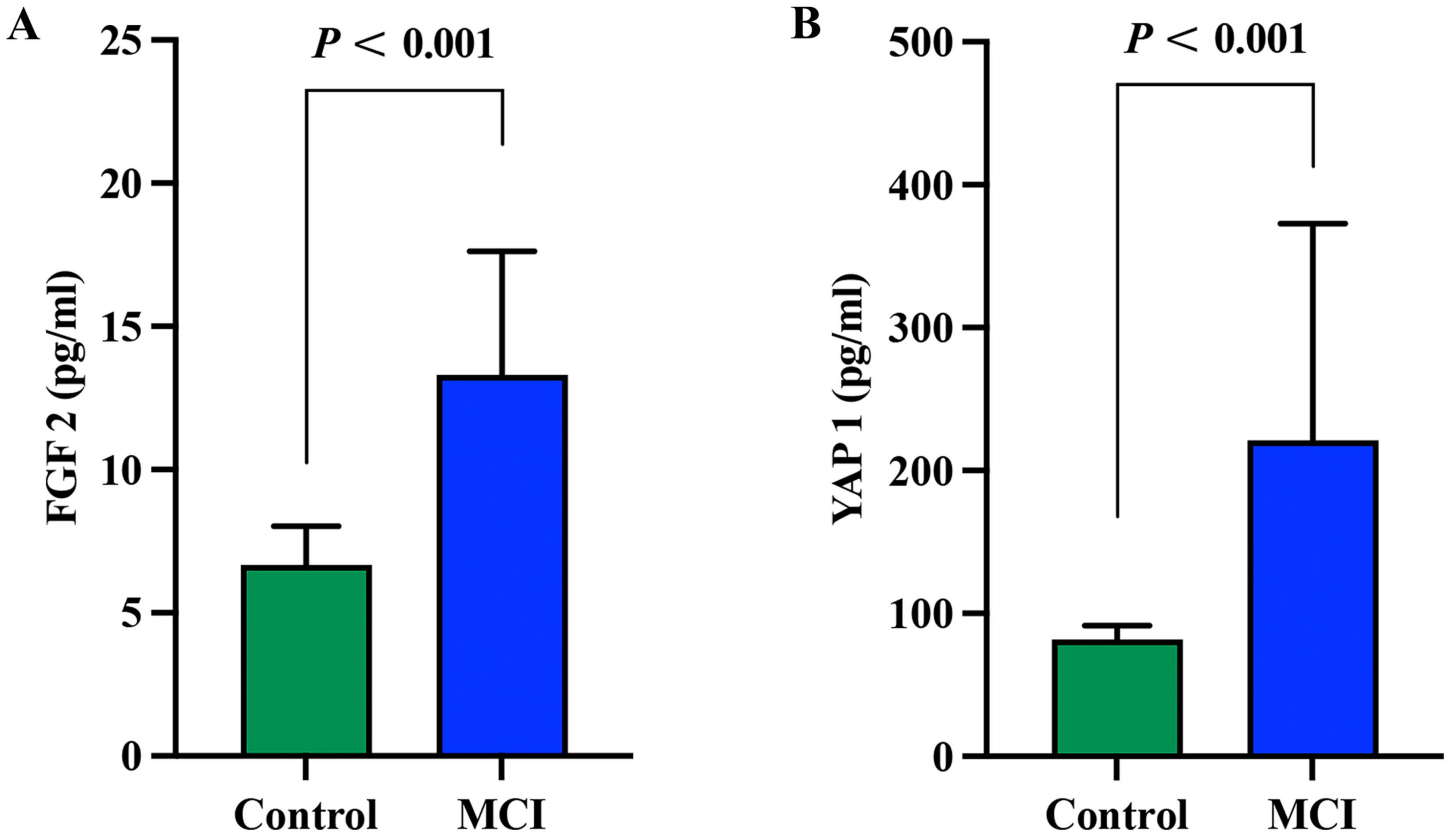
Comparison of plasma FGF2 and YAP1 expression levels between the MCI group and the control group. **(A)** Comparison of FGF2 expression levels between the MCI group and the control group. **(B)** Comparison of YAP1 expression levels between the MCI group and the control group. Intergroup comparisons were both analyzed using the Mann-Whitney U test, with a *p* value < 0.05 considered statistically significant. MCI, Mild cognitive impairment.

As shown in Figure 2, the plasma FGF2 activity of the control group and MCI group were 6.65 [5.77–8.01]pg./ml and 13.31 [10.09–17.63]pg./ml, respectively. The plasma YAP1 activity of the control group and MCI group were 81.93 [67.25–91.54]pg./ml and 221.04 [144.05–362.96]pg./ml, respectively. The expression levels of plasma FGF2 and YAP1 proteins in the MCI group were significantly increased compared to the control group (*p* < 0.001 for both).

### 3.5 Correlation analysis between FGF2, YAP1 and cognitive function

Spearman correlation analyses were performed to explore the relationship between FGF2, YAP1 activity and different dimensions of cognitive function, as shown in Table 2. The expression level of plasma FGF2 was positively correlated with the occurrence of MCI, and significantly negatively correlated with MoCA score, visuospatial executive, attention, language, abstraction and delayed recall score. The expression level of plasma YAP1 was significantly negatively correlated with global cognitive function or MoCA score, and was significantly negatively correlated with all cognitive function dimensions scores, except for orientation ability.

**Table 2:** Correlation analysis between FGF2, YAP1 and cognitive function.

Parameter	FGF2		YAP1	
	*r*	*p*	*r*	*p*
MCI	0.626	<0.001	0.726	<0.001
MoCA score	−0.404	<0.001	−0.591	<0.001
Visuospatial Executive	−0.220	0.002	−0.297	<0.001
Naming	−0.185	0.009	−0.150	0.034
Attention	−0.049	0.488	−0.269	<0.001
Language	−0.178	0.012	−0.259	<0.001
Abstraction	−0.234	<0.001	−0.215	0.002
Delayed Recall	−0.323	<0.001	−0.481	<0.001
Orientation	−0.023	0.748	−0.071	0.317

MCI, Mild cognitive impairment; MoCA, Montreal Cognitive Assessment.

### 3.6 Diagnostic prediction efficacy of FGF2 and YAP1 for MCI

To further investigate whether plasma FGF2 and YAP1 activity maybe independently associated with MCI in the elderly. We constructed a multivariate logistic regression model (Table 3). Adjusting for confounding factors did not change the association between plasma FGF2 and YAP1 expression levels and the occurrence of MCI. For every 1 unit increase in the expression level of FGF2 or YAP1 expression level, the incidence of MCI increases by 1.843 times and 1.086 times, respectively, indicating that high plasma FGF2 and YAP1 expression levels were independently associated with the occurrence of MCI in the elderly.

**Table 3:** Multivariate analysis of parameters associated between FGF2 and MCI.

Model	FGF2				YAP1			
	*p*	B	Exp(B)	95% CI	*p*	B	Exp(B)	95% CI
Model 1	<0.001	0.556	1.744	1.464–2.078	<0.001	0.076	1.079	1.051–1.108
Model 2	<0.001	0.558	1.746	1.465–2.083	<0.001	0.078	1.081	1.052–1.111
Model 3	<0.001	0.551	1.734	1.455–2.067	<0.001	0.078	1.081	1.052–1.111
Model 4	<0.001	0.577	1.781	1.477–2.148	<0.001	0.077	1.081	1.051–1.111
Model 5	<0.001	0.611	1.843	1.511–2.248	<0.001	0.082	1.086	1.052–1.120

Model 1: crude model. Model 2: adjusted for age and sex. Model 3: adjusted for model 2 covariates + Education and BMI. Model 4: adjusted for model 3 covariates + Occupation type, Household income, Marital status, and Residency status. Model 5: adjusted for model 4 covariates + Past history. *p* < 0.05 indicates significance.

**Figure 3:**
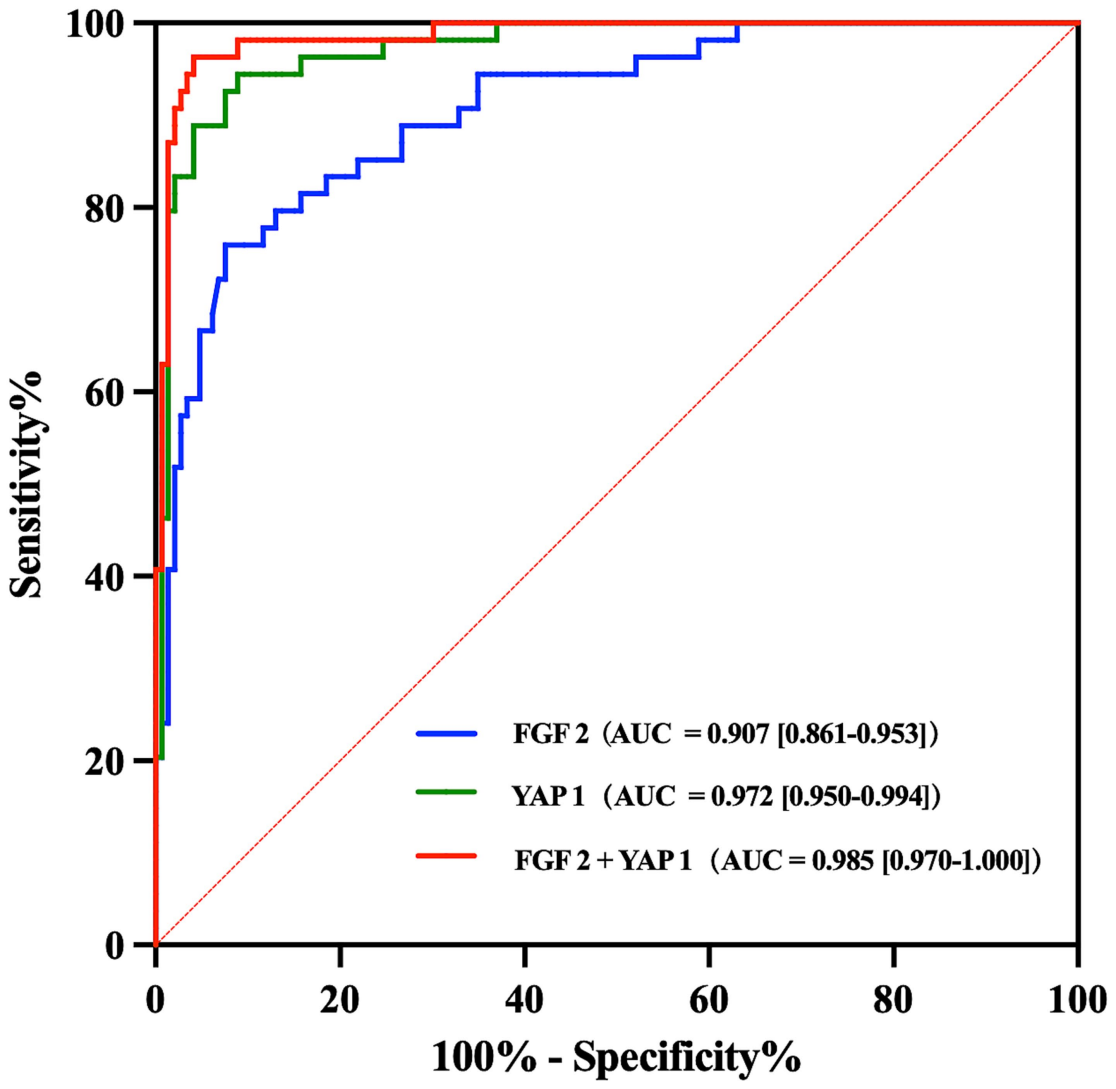
ROC curve analyses of FGF2 and YAP1 for predicting MCI. AUC and 95% CI were also calculated for each model. ROC, Receiver operating characteristic; MCI, Mild cognitive impairment; AUC, Area under the curve; CI, Confidence interval.

The discriminatory abilities of FGF2 and YAP1 were assessed by calculating the AUC and plotting the ROC curve for predictive models, as shown in Figure 3. The AUC of FGF2 model and YAP1 model were 0.907 (95% CI = 0.861–0.953) and 0.972 (95% CI = 0.950–0.994), respectively, indicating that both FGF2 and YAP1 had excellent predictive and discriminative power for MCI. In addition, the result suggested that the combined detection of FGF2 and YAP1 expression levels can further improve the predictive performance of the model for MCI.

## 4 Discussion

Early detection of MCI or preclinical dementia and providing early prevention or intervention for dementia are crucial. However, AD is still largely a clinical diagnosis at present, although Aβ in the brain begins decades before symptoms appear. According to reports, Symptomatic AD is misdiagnosed in 25 to 35% of patients treated at specialized clinics and likely even more patients treated in primary care (Rabinovici et al., 2019; Cheslow et al., 2024). Identifying effective biomarkers that can provide timely and accurate diagnosis will enhance the ability greatly to prevent cognitive decline in AD (Reiman et al., 2012). PET or detection of CSF biomarkers can aid in the diagnosis of AD, but these are difficult to popularize due to drawbacks such as high cost, low flux, radiation exposure, and invasiveness. Blood biomarkers are more time-saving, economical, and convenient for patients, which may replace CSF tests and PET, greatly promoting the early diagnosis and treatment of AD.

Our bioinformatic analysis in the present study showed that in the GSE29378 and GSE12685 datasets, FGF2 and YAP1 genes were significantly upregulated in the AD group compared to the control group. Clinical trial validation showed that the expression levels of plasma FGF2 and YAP1 proteins in the MCI group were significantly increased compared to the control group. The regression model confirmed significant correlations between plasma FGF2 and YAP1 expression levels and the occurrence of MCI, even after adjusting for confounding factors, indicating that high plasma FGF2 and YAP1 expression levels were independently associated with the occurrence of MCI in elderly individuals, and the discriminative power was excellent confirmed by ROC analyses. Previous studies have demonstrated that plasma p-tau measurements exhibited high diagnostic accuracy in distinguishing AD from other neurodegenerative disorders, with this validity supported by postmortem neuropathological investigations (Karikari et al., 2020; Palmqvist et al., 2020; Ashton et al., 2021). In particular, plasma p-tau217 effectively differentiated AD from non-AD cases, with an AUC of 0.89 (95% CI = 0.81–0.97; Palmqvist et al., 2020). Our findings confirmed that the performance of plasma FGF2 and YAP1 was comparable to that of previously reported traditional plasma biomarkers, underscoring substantial exploratory potential.

Emerging evidence has increasingly demonstrated that ferroptosis contributes to neuronal cell death in the brain, particularly within brain regions implicated in AD (Wang B. et al., 2022). Through functional enrichment analysis focusing on ferroptosis, we identified a close association between FGF2, YAP1, and MCI. Consequently, it is plausible to hypothesize that FGF2 and YAP1 may exert regulatory effects on the pathogenesis and progression of AD via the mediation of ferroptosis. Previous studies have confirmed that both FGF2 and YAP1 exert antioxidative, anti-apoptotic, and tissue-protective effects by regulating ferroptosis (Chen F. et al., 2023; Furutake et al., 2024). Specifically, Chen et al. demonstrated that FGF2 protects endothelial cells via KLF2-mediated ferroptosis inhibition (Chen R. et al., 2023), while YAP1 reduces oxidative stress and inflammation, and promotes cell survival in diseases like acute lung injury (Zhang et al., 2022) and osteoarthritis (Chen F. et al., 2023; Chen R. et al., 2023). In AD, preliminary evidence has shown FGF2 improved cognition by modulating ferroptosis and supporting neuronal survival (Li et al., 2025), whereas YAP1 mitigates cognitive deficits and reverses AD pathology via CDK6 signaling (Xu et al., 2021) or artemisinin activation (Zhou et al., 2023). Accordingly, integrating prior literature with the findings of the present study, it is hypothesized that FGF2 and YAP1 may exert cognitive-improving effects through the mediation of ferroptosis, a pivotal pathogenic mechanism in AD, presumably via the attenuation of neuroinflammation, oxidative stress, and mitochondrial dysfunction, although this necessitates further in-depth exploration and validation (Wang F. et al., 2022).

FGFs are a superfamily of proteins, most of which bind heparin and extracellular heparin sulfate proteoglycans (HSPGs) and have a homologous central core of 140 amino acids. FGF2 has been proven to have pleiotropic effects in different tissues and organs, including potent angiogenesis and important roles in the differentiation and function of the central nervous system (Woodbury and Ikezu, 2014). Previous researchers have found that FGF2 is widely involved in various BP, including cell proliferation, differentiation, migration, and survival, and plays a critical role in the nervous system, contributing to neurodevelopment, repair, and protection (Wang et al., 2020; Lebowitz et al., 2021, 2023). For instance, Lamus et al. (2020) found that FGF2/EGF promotes the proliferation and neurogenesis of rat embryonic brain neural epithelial precursor cells. Upadhya et al. (2020) demonstrated that extracellular vesicles shed from immature astrocytes contain numerous neuroprotective compounds, including FGF2 and vascular endothelial growth factor. In the nervous system, FGF2 promotes neuronal growth and survival, having a positive role in neuroprotection (Masgutov et al., 2021). Contrary to successful reports of FGF2 in neuroprotection and neurogenesis, the elevation of FGF2 in AD brain, especially in the hippocampus, has been found (Stopa et al., 1990; Hock et al., 2000). Animal experiments have shown that serum adenovirus associated 2/1 mediates FGF2 gene expression in AD mouse models, promotes neuronal proliferation in the hippocampal cornu ammonis 1 and subventricular zone regions, and enhances spatial learning ability in mice (Kiyota et al., 2011). Therefore, FGF2 shows potential diagnostic and therapeutic value for AD. However, previous studies have mostly focused on animal experiments or changes in FGF2 levels in the brain. The relationship between the expression level of FGF2 in human plasma and AD has not been determined yet, and how the expression level of FGF2 changes in the elderly population with MCI is even more unclear. In the present study, we found the expression level of FGF2 protein in the plasma of elderly people with MCI was significantly higher than that of the control group, and confirmed that plasma FGF2 was an independent risk factor for the occurrence of MCI in the elderly. The novel biomarker with minimally invasive, easily obtainable, and cost-effective provides new insights for the early screening and diagnosis of MCI in the elderly.

We also found that the expression level of plasma YAP1 protein was closely related to the occurrence of MCI, and the discriminative power was excellent. YAP1, namely yes-associated protein 1, is a key effector in the Hippo signaling pathway, also known as YAP or YAP65. It has been proved that YAP1 had an active role in regulating neural precursor proliferation, neuronal specification, and neocortical astrocytic differentiation (Fernandez-L et al., 2009; Musah et al., 2014; Huang et al., 2016). Previous experiments revealed that YAP was down-regulated and inactivated in hippocampal astrocytes of AD model mice in a hippocampal pathway-dependent manner (Ries et al., 2016). Xu et al. (2018) demonstrated that knockdown of YAP1 expression leaded to increased Ab production and tau phosphorylation in U251-APP cells, whereas overexpression of YAP1 had opposite effects. Additionally, in studies investigating the cognitive improvement induced by dexmedetomidine in AD patients, the miR-129/YAP1/JAG1 axis was identified as a potential mechanism underlying dexmedetomidine-mediated protection against cognitive impairment (Sun et al., 2020). Collectively, these findings indicate that YAP1 may serve as a potential biomarker for AD and a protective regulator of cognitive function, consistent with the results of the present study. However, interestingly, in contrast to the downregulation of YAP1 gene expression observed in cellular experiments, our study revealed a significant elevation in plasma YAP1 protein levels among MCI patients. Previous studies have identified YAP1 as the most critical hub molecule in the AD molecular network, further noting that the upregulation of YAP1 mRNA they observed may represent a protective transcriptional response aimed at compensating for reduced YAP1 protein levels (Xu et al., 2018). Concurrently, animal studies by Ries et al. (2016) demonstrated that activation of the YAP-CDK6 pathway ameliorates cognitive function in both AD model mice and senescent mice. Moreover, Tanaka et al. (2020) provided evidence that neuronal necrosis induced by YAP deprivation occurs most actively in the preclinical AD and MCI. Drawing on these lines of evidence, we hypothesize that YAP1 may act as a compensatory neuroprotective factor generated during the onset of cognitive impairment. Specifically, in the early stages of cognitive decline in MCI, the compensatory elevation of plasma YAP1 protein levels may represent a protective response mechanism against the progression of cognitive dysfunction. Nevertheless, the precise regulatory mechanisms governing YAP1 expression and its biological properties in mediating the progression of cognitive impairment warrant further investigation.

Despite the rigorous bioinformatics analysis and clinical trial validation work, there were still some limitations in the present study. First, although it has been demonstrated that the expression levels of FGF2 and YAP1 in plasma were upregulated in elderly individuals with MCI, the specific pathways of action and regulatory mechanisms in the pathogenesis of AD still needs to be clarified in the future. Second, this study was a single-center research with a relatively small sample size, and the subjects were all Chinese. Therefore, the conclusions may not be applicable to other ethnic groups, and future validation across diverse cohorts is imperative to strengthen the generalizability of these findings. Based on the findings of this study, experimental models will be established to investigate the mechanisms of AD regulation by these molecules, and we will also expand the clinical sample size to include subjects with different levels of cognitive function, in order to explore the role of these novel biomarkers in predicting AD progression in clinical practice, promote the development of minimally invasive and convenient early screening tools.

## 5 Conclusion

In the present study, the high expression of plasma FGF2 and YAP1 proteins can served as independent predictive risk factors for MCI in the elderly. Our findings may provide targets for the development of early minimally invasive and convenient screening tools for MCI, and even for the treatment of AD in the future.

## Data Availability

The original contributions presented in the study are included in the article/Supplementary material, further inquiries can be directed to the corresponding authors.

## Footnotes

^1^ https://string-db.org/

^2^ http://www.genome.jp

^3^ http://www.broadinstitute.org/gsea/downloads.jsp

## Glossary

AD: Alzheimer’s disease
MCI: Mild cognitive impairment
FGF2: Fibroblast growth factor 2
YAP1: Yes-associated protein 1
SOX2: SRY-Box transcription factor 2
FLNC: Filamin C
GJA1: Gap junction protein alpha 1
DCLK1: Doublecortin like kinase 1
SOX9: SRY-Box transcription Factor 9
Aβ: Amyloid beta
PET: Positron emission tomography
p-tau181: phosphorylated tau181
CSF: Cerebrospinal fluid
GFAP: Glial fibrillary acidic protein
GEO: Gene expression omnibus
DEGs: Differentially expressed genes
PCA: Principal component analysis
FC: Fold changes
FDR: False discovery rate
WGCNA: Weighted gene co-expression network analysis
TOM: Topological overlap measure
PPI: Protein protein interaction
STRING: Search tool for the retrieval of interacting genes
GO: Gene ontology
BP: Biological processes
CC: Cellular components
MF: Molecular functions
KEGG: Kyoto encyclopedia of genes and genomes
GSEA: Gene set enrichment analysis
CTD: Comparative toxicogenomics Database
MoCA: Montreal cognitive assessment
BMI: Body mass index
SPPB: Short physical performance battery
ELISA: Enzyme linked immunosorbent assay
OD: Optical density
SD: Standard deviation
ROC: Receiver operating characteristic
AUC: Area under the curve
CI: Confidence interval
HSPGs: Heparin sulfate proteoglycans

## References

[ref1] Albert M. S. DeKosky S. T. Dickson D. Dubois B. Feldman H. H. Fox N. C.. (2011). The diagnosis of mild cognitive impairment due to Alzheimer's disease: recommendations from the National Institute on Aging-Alzheimer's Association workgroups on diagnostic guidelines for Alzheimer's disease. Alzheimers Dement. 7, 270–279. doi: 10.1016/j.jalz.2011.03.008 21514249 PMC3312027

[ref2] Ashton N. J. Pascoal T. A. Karikari T. K. Benedet A. L. Lantero-Rodriguez J. Brinkmalm G.. (2021). Plasma p-tau231: a new biomarker for incipient Alzheimer's disease pathology. Acta Neuropathol. 141, 709–724. doi: 10.1007/s00401-021-02275-6 33585983 PMC8043944

[ref3] Chen R. Ying C. Zou Y. Lin C. Fu Q. Xiang Z.. (2023). Sarsasapogenin inhibits YAP1-dependent chondrocyte ferroptosis to alleviate osteoarthritis. Biomed. Pharmacother. 168:115772. doi: 10.1016/j.biopha.2023.115772 37879209

[ref4] Chen F. Zhan J. Liu M. Mamun A. A. Huang S. Tao Y.. (2023). FGF2 alleviates microvascular ischemia-reperfusion injury by KLF2-mediated Ferroptosis inhibition and antioxidant responses. Int. J. Biol. Sci. 19, 4340–4359. doi: 10.7150/ijbs.85692 37705747 PMC10496511

[ref5] Cheslow L. Snook A. E. Waldman S. A. (2024). Biomarkers for managing neurodegenerative diseases. Biomolecules 14:398. doi: 10.3390/biom14040398 38672416 PMC11048498

[ref6] GBDS Collaborators (2022). Estimation of the global prevalence of dementia in 2019 and forecasted prevalence in 2050: an analysis for the global burden of disease study 2019. Lancet Public Health 7, e105–e125. doi: 10.1016/S2468-2667(21)00249-8 34998485 PMC8810394

[ref7] Fernandez-L A. Northcott P. A. Dalton J. Fraga C. Ellison D. Angers S.. (2009). YAP1 is amplified and up-regulated in hedgehog-associated medulloblastomas and mediates sonic hedgehog-driven neural precursor proliferation. Genes Dev. 23, 2729–2741. doi: 10.1101/gad.1824509 19952108 PMC2788333

[ref8] Furutake Y. Yamaguchi K. Yamanoi K. Kitamura S. Takamatsu S. Taki M.. (2024). YAP1 suppression by ZDHHC7 is associated with Ferroptosis resistance and poor prognosis in ovarian clear cell carcinoma. Mol. Cancer Ther. 23, 1652–1665. doi: 10.1158/1535-7163.MCT-24-0145 38958503

[ref9] Gustavsson A. Norton N. Fast T. Frölich L. Georges J. Holzapfel D.. (2023). Global estimates on the number of persons across the Alzheimer's disease continuum. Alzheimers Dement. 19, 658–670. doi: 10.1002/alz.12694 35652476

[ref10] Hambright W. S. Fonseca R. S. Chen L. Na R. Ran Q. (2017). Ablation of ferroptosis regulator glutathione peroxidase 4 in forebrain neurons promotes cognitive impairment and neurodegeneration. Redox Biol. 12, 8–17. doi: 10.1016/j.redox.2017.01.021 28212525 PMC5312549

[ref11] Hansson O. (2021). Biomarkers for neurodegenerative diseases. Nat. Med. 27, 954–963. doi: 10.1038/s41591-021-01382-x 34083813

[ref12] Hansson O. Blennow K. Zetterberg H. Dage J. (2023). Blood biomarkers for Alzheimer's disease in clinical practice and trials. Nature Aging 3, 506–519. doi: 10.1038/s43587-023-00403-3 37202517 PMC10979350

[ref13] Hock C. Heese K. Hulette C. Rosenberg C. Otten U. (2000). Region-specific neurotrophin imbalances in Alzheimer disease: decreased levels of brain-derived neurotrophic factor and increased levels of nerve growth factor in hippocampus and cortical areas. Arch. Neurol. 57, 846–851. doi: 10.1001/archneur.57.6.846 10867782

[ref14] Huang Z. Hu J. Pan J. Wang Y. Hu G. Zhou J.. (2016). YAP stabilizes SMAD1 and promotes BMP2-induced neocortical astrocytic differentiation. Development 143, 2398–2409. doi: 10.1242/dev.130658 27381227 PMC4958318

[ref15] Jia J. Wei C. Chen S. Li F. Tang Y. Qin W.. (2018). The cost of Alzheimer's disease in China and re-estimation of costs worldwide. Alzheimers Dement. 14, 483–491. doi: 10.1016/j.jalz.2017.12.006 29433981

[ref16] Karikari T. K. Pascoal T. A. Ashton N. J. Janelidze S. Benedet A. L. Rodriguez J. L.. (2020). Blood phosphorylated tau 181 as a biomarker for Alzheimer's disease: a diagnostic performance and prediction modelling study using data from four prospective cohorts. Lancet Neurol. 19, 422–433. doi: 10.1016/S1474-4422(20)30071-5 32333900

[ref17] Kiyota T. Ingraham K. L. Jacobsen M. T. Xiong H. Ikezu T. (2011). FGF2 gene transfer restores hippocampal functions in mouse models of Alzheimer's disease and has therapeutic implications for neurocognitive disorders. Proc. Natl. Acad. Sci. USA 108, E1339–E1348. doi: 10.1073/pnas.1102349108 22042871 PMC3241747

[ref18] Kwiatek-Majkusiak J. Dickson D. W. Tacik P. Aoki N. Tomasiuk R. Koziorowski D.. (2015). Relationships between typical histopathological hallmarks and the ferritin in the hippocampus from patients with Alzheimer's disease. Acta Neurobiol. Exp. (Wars) 75, 391–398. doi: 10.55782/ane-2015-2044 26994418

[ref19] Lamus F. Martín C. Carnicero E. Moro J. A. Fernández J. Mano A.. (2020). FGF2/EGF contributes to brain neuroepithelial precursor proliferation and neurogenesis in rat embryos: the involvement of embryonic cerebrospinal fluid. Develop. Dynamics: Official Pub. American Assoc. 249, 141–153. doi: 10.1002/dvdy.135 31736174

[ref20] Lebowitz E. R. Marin C. E. Orbach M. Salmaso N. Vaccarino F. M. Silverman W. K. (2023). Maternal FGF2 levels associated with child anxiety and depression symptoms through child FGF2 levels. J. Affect. Disord. 326, 193–197. doi: 10.1016/j.jad.2023.01.090 36717031 PMC10104478

[ref21] Lebowitz E. R. Orbach M. Marin C. E. Salmaso N. Vaccarino F. M. Silverman W. K. (2021). Fibroblast growth factor 2 implicated in childhood anxiety and depression symptoms. J. Affect. Disord. 282, 611–616. doi: 10.1016/j.jad.2020.12.055 33445083 PMC7897422

[ref22] Li Y. Yang C. Liu X. Shu J. Zhao N. Sun Z.. (2025). Potential therapeutic targets for Alzheimer's disease: fibroblast growth factors and their regulation of ferroptosis, pyroptosis and autophagy. Neuroscience 573, 42–51. doi: 10.1016/j.neuroscience.2025.03.009 40096963

[ref23] Masgutov R. Zeinalova A. Bogov A. Masgutova G. Salafutdinov I. Garanina E.. (2021). Angiogenesis and nerve regeneration induced by local administration of plasmid pBud-coVEGF165-coFGF2 into the intact rat sciatic nerve. Neural Regen. Res. 16, 1882–1889. doi: 10.4103/1673-5374.306090 33510097 PMC8328758

[ref24] Musah S. Wrighton P. J. Zaltsman Y. Zhong X. Zorn S. Parlato M. B.. (2014). Substratum-induced differentiation of human pluripotent stem cells reveals the coactivator YAP is a potent regulator of neuronal specification. Proc. Natl. Acad. Sci. USA 111, 13805–13810. doi: 10.1073/pnas.1415330111 25201954 PMC4183276

[ref25] Palmqvist S. Janelidze S. Quiroz Y. T. Zetterberg H. Lopera F. Stomrud E.. (2020). Discriminative accuracy of plasma Phospho-tau217 for Alzheimer disease vs other neurodegenerative disorders. JAMA 324, 772–781. doi: 10.1001/jama.2020.12134 32722745 PMC7388060

[ref26] Petersen R. C. (2004). Mild cognitive impairment as a diagnostic entity. J. Intern. Med. 256, 183–194. doi: 10.1111/j.1365-2796.2004.01388.x 15324362

[ref27] Rabinovici G. D. Gatsonis C. Apgar C. Chaudhary K. Gareen I. Hanna L.. (2019). Association of Amyloid Positron Emission Tomography with Subsequent Change in clinical management among Medicare beneficiaries with mild cognitive impairment or dementia. JAMA 321, 1286–1294. doi: 10.1001/jama.2019.2000 30938796 PMC6450276

[ref28] Reiman E. M. Quiroz Y. T. Fleisher A. S. Chen K. Velez-Pardo C. Jimenez-Del-Rio M.. (2012). Brain imaging and fluid biomarker analysis in young adults at genetic risk for autosomal dominant Alzheimer's disease in the presenilin 1 E280A kindred: a case-control study. Lancet Neurol. 11, 1048–1056. doi: 10.1016/S1474-4422(12)70228-4 23137948 PMC4181671

[ref29] Ries M. Loiola R. Shah U. N. Gentleman S. M. Solito E. Sastre M. (2016). The anti-inflammatory Annexin A1 induces the clearance and degradation of the amyloid-β peptide. J. Neuroinflammation 13:234. doi: 10.1186/s12974-016-0692-6 27590054 PMC5010757

[ref30] Rissman R. A. Langford O. Raman R. Donohue M. C. Abdel-Latif S. Meyer M. R.. (2024). Plasma Aβ42/Aβ40 and phospho-tau217 concentration ratios increase the accuracy of amyloid PET classification in preclinical Alzheimer's disease. Alzheimers Dement. 20, 1214–1224. doi: 10.1002/alz.13542 37932961 PMC10916957

[ref31] Ryu J. C. Zimmer E. R. Rosa-Neto P. Yoon S. O. (2019). Consequences of metabolic disruption in Alzheimer's disease pathology. Neurotherapeutics 16, 600–610. doi: 10.1007/s13311-019-00755-y 31270743 PMC6694332

[ref32] Stopa E. G. Gonzalez A. M. Chorsky R. Corona R. J. Alvarez J. Bird E. D.. (1990). Basic fibroblast growth factor in Alzheimer's disease. Biochem. Biophys. Res. Commun. 171, 690–696. doi: 10.1016/0006-291x(90)91201-3 2403357

[ref33] Sun W. Zhao J. Li C. (2020). Dexmedetomidine provides protection against hippocampal neuron apoptosis and cognitive impairment in mice with Alzheimer's disease by mediating the miR-129/YAP1/JAG1 Axis. Mol. Neurobiol. 57, 5044–5055. doi: 10.1007/s12035-020-02069-z 32839917

[ref34] Tanaka H. Homma H. Fujita K. Kondo K. Yamada S. Jin X.. (2020). YAP-dependent necrosis occurs in early stages of Alzheimer's disease and regulates mouse model pathology. Nat. Commun. 11:507. doi: 10.1038/s41467-020-14353-6 31980612 PMC6981281

[ref35] Upadhya R. Zingg W. Shetty S. Shetty A. K. (2020). Astrocyte-derived extracellular vesicles: Neuroreparative properties and role in the pathogenesis of neurodegenerative disorders. J. Controlled Release: Official J. Controlled Release 323, 225–239. doi: 10.1016/j.jconrel.2020.04.017 PMC7299747 32289328

[ref36] Vermunt L. Sikkes S. van den Hout A. Handels R. Bos I. van der Flier W. M.. (2019). Duration of preclinical, prodromal, and dementia stages of Alzheimer's disease in relation to age, sex, and APOE genotype. Alzheimers Dement. 15, 888–898. doi: 10.1016/j.jalz.2019.04.001 31164314 PMC6646097

[ref37] Wang B. Fu C. Wei Y. Xu B. Yang R. Li C.. (2022). Ferroptosis-related biomarkers for Alzheimer's disease: identification by bioinformatic analysis in hippocampus. Front. Cell. Neurosci. 16:1023947. doi: 10.3389/fncel.2022.1023947 36467613 PMC9709107

[ref38] Wang J. Shu H. Guo S. (2020). MiR-646 suppresses proliferation and metastasis of non-small cell lung cancer by repressing FGF2 and CCND2. Cancer Med. 9, 4360–4370. doi: 10.1002/cam4.3062 32347652 PMC7300416

[ref39] Wang F. Wang J. Shen Y. Li H. Rausch W. D. Huang X. (2022). Iron Dyshomeostasis and Ferroptosis: a new Alzheimer's disease hypothesis. Front. Aging Neurosci. 14:830569. doi: 10.3389/fnagi.2022.830569 35391749 PMC8981915

[ref40] Woodbury M. E. Ikezu T. (2014). Fibroblast growth factor-2 signaling in neurogenesis and neurodegeneration. J. Neuroimmune Pharmacol.: Official J. Society 9, 92–101. doi: 10.1007/s11481-013-9501-5 PMC4109802 24057103

[ref41] Xu X. Shen X. Wang J. Feng W. Wang M. Miao X.. (2021). YAP prevents premature senescence of astrocytes and cognitive decline of Alzheimer's disease through regulating CDK6 signaling. Aging Cell 20:e13465. doi: 10.1111/acel.13465 34415667 PMC8441453

[ref42] Xu M. Zhang D. F. Luo R. Wu Y. Zhou H. Kong L. L.. (2018). A systematic integrated analysis of brain expression profiles reveals YAP1 and other prioritized hub genes as important upstream regulators in Alzheimer's disease. Alzheimers Dement. 14, 215–229. doi: 10.1016/j.jalz.2017.08.012 28923553

[ref43] Yan N. Zhang J. (2019). Iron metabolism, Ferroptosis, and the links with Alzheimer's disease. Front. Neurosci. 13:1443. doi: 10.3389/fnins.2019.01443 32063824 PMC7000453

[ref44] Zhang J. Zheng Y. Wang Y. Wang J. Sang A. Song X.. (2022). YAP1 alleviates sepsis-induced acute lung injury via inhibiting ferritinophagy-mediated ferroptosis. Front. Immunol. 13:884362. doi: 10.3389/fimmu.2022.884362 35979359 PMC9376389

[ref45] Zhou W. Lei B. Yang C. Silva M. Xing X. Yu H.. (2023). *Artemisia annua* extract improves the cognitive deficits and reverses the pathological changes of Alzheimer's disease via regulating YAP signaling. Int. J. Mol. Sci. 24:5259. doi: 10.3390/ijms24065259 36982332 PMC10049624

