# Supplementary material for: Plasma FGF2 and YAP1 as novel biomarkers for MCI in the elderly: analysis via bioinformatics and clinical study
Source: Front Neurosci. 2025 Aug 26;19:1663276. doi: 10.3389/fnins.2025.1663276 (PMC12417436; doi:10.3389/fnins.2025.1663276)
Supplement: Supplementary file 1 [file Supplementary_file_1.docx]

Supplementary materials

# 1 Supplementary Figures and tables

## 1.1 Supplementary Figures


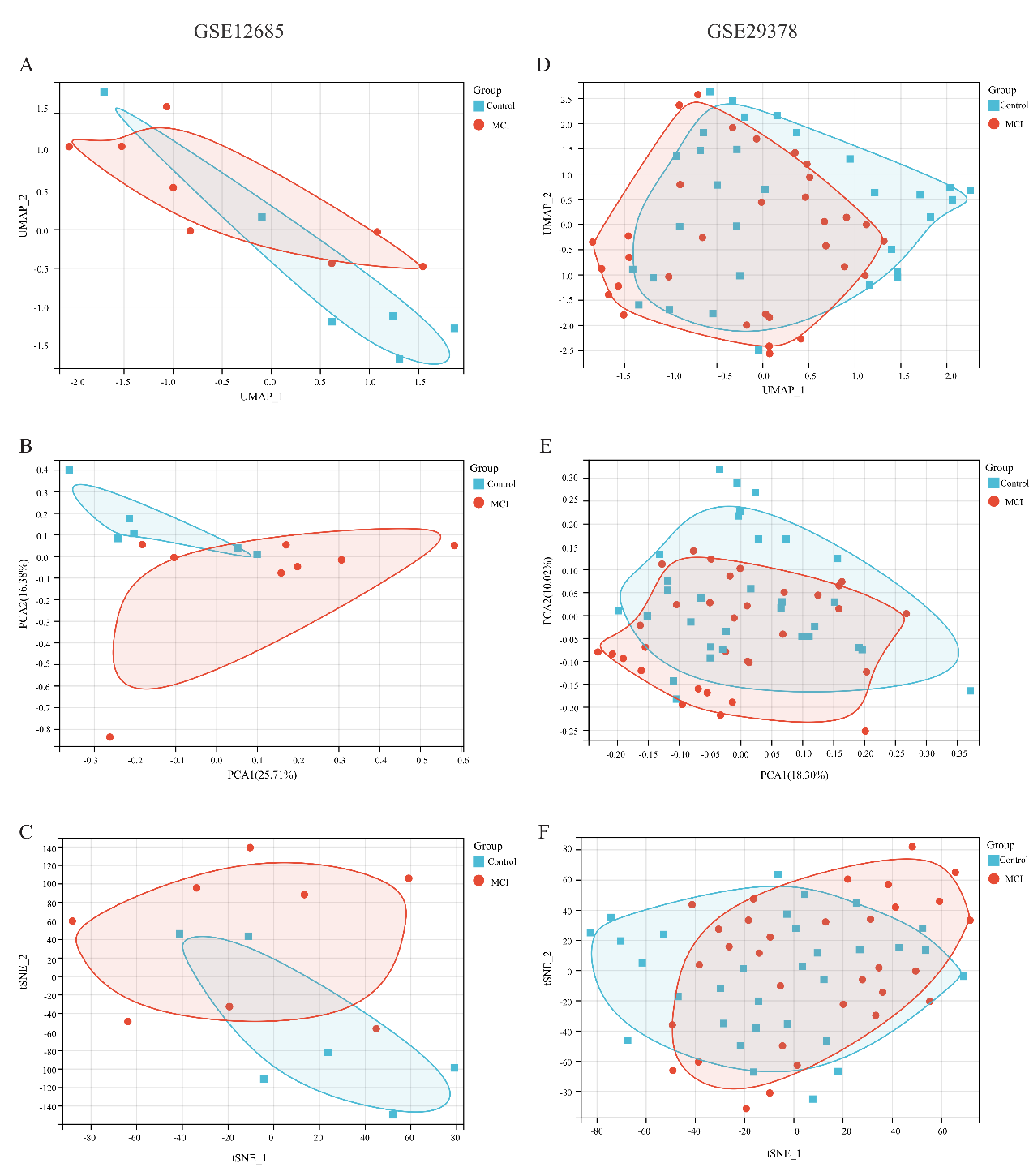
**Supplementary Figure 1.** Principal component analysis. (A) UMAP analysis result of GSE12685; (B) PCA analysis results of GSE12685; (C) tSNE analysis results of GSE12685; (D) UMAP analysis result of GSE29378; (E) PCA analysis results of GSE29378; (F) tSNE analysis results of GSE29378.


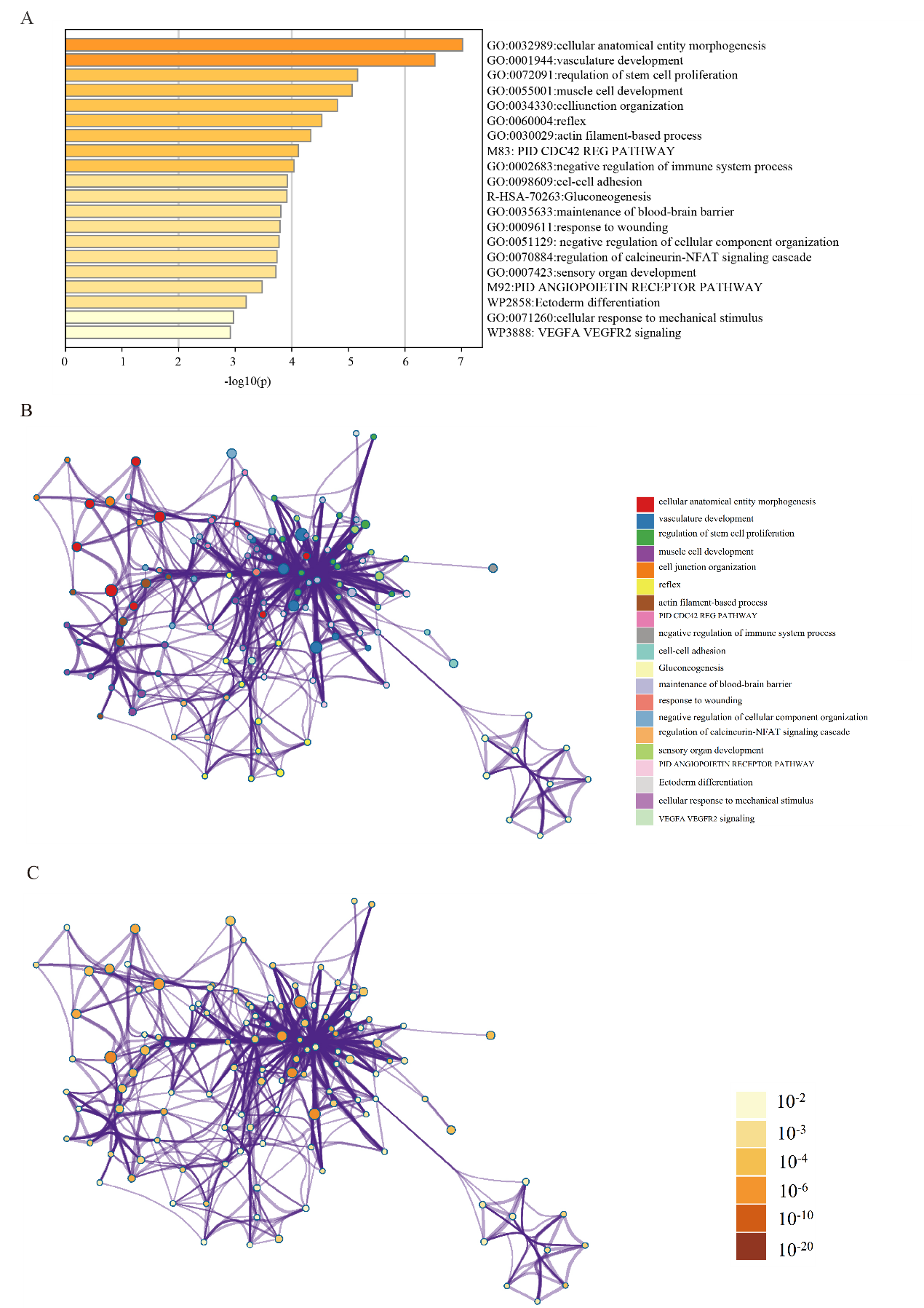
**Supplementary Figure 2.** Metascape enrichment analysis. (A) Bar graph of enriched terms across input gene lists, colored by P-values; (B) Network of enriched terms: colored by cluster identification number, where nodes that share the same cluster identification number are typically close to each other; (C) Colored by P-value, where terms containing more genes tend to have a more significant P-value.


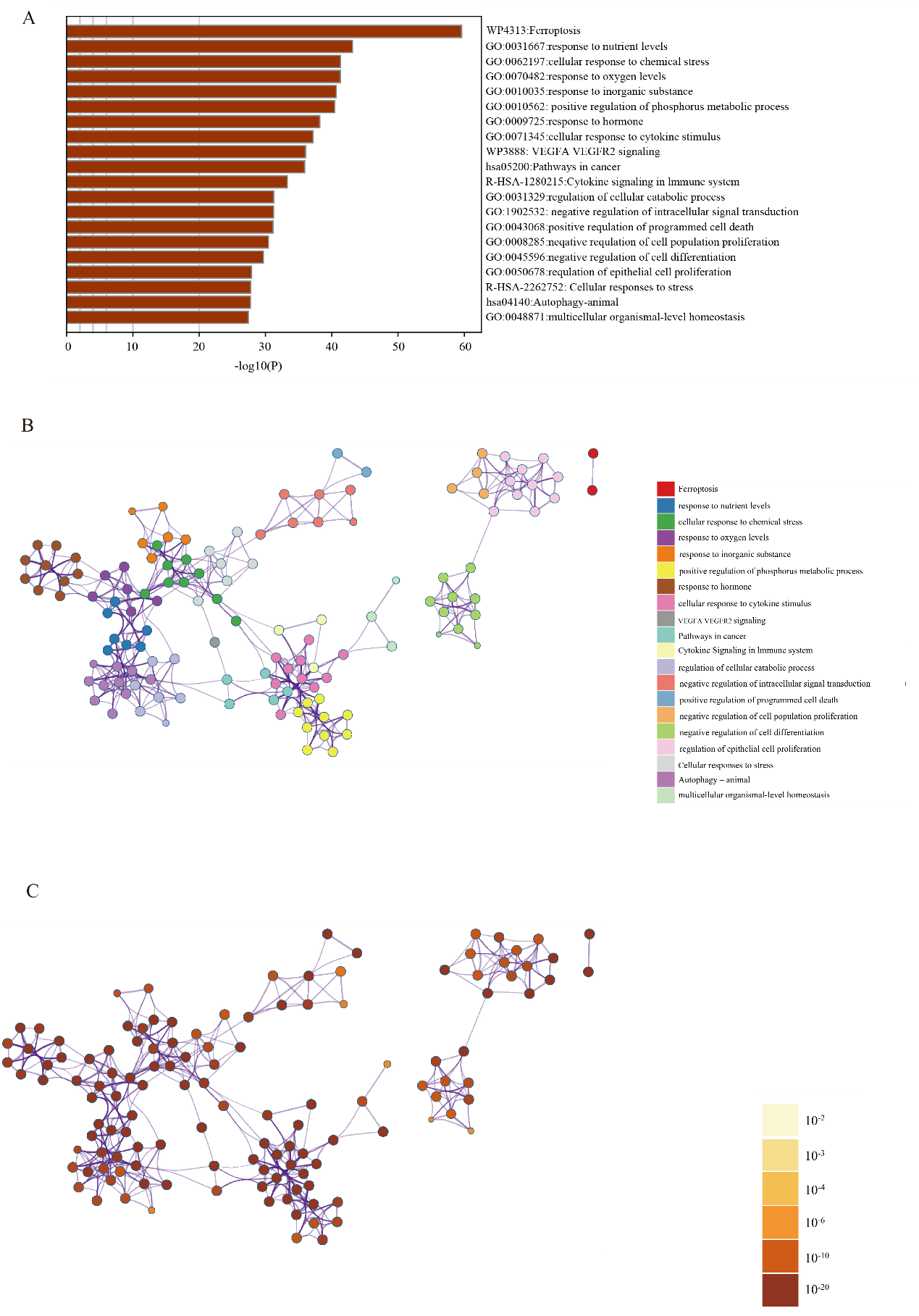


**Supplementary Figure 3.** Metascape enrichment analysis of ferroptosis. (A) Bar graph of enriched terms across input gene lists, colored by P-values; (B) Network of enriched terms: colored by cluster identification number, where nodes that share the same cluster ID are typically close to each other; (C) Colored by P-value, where terms containing more genes tend to have a more significant P-value.


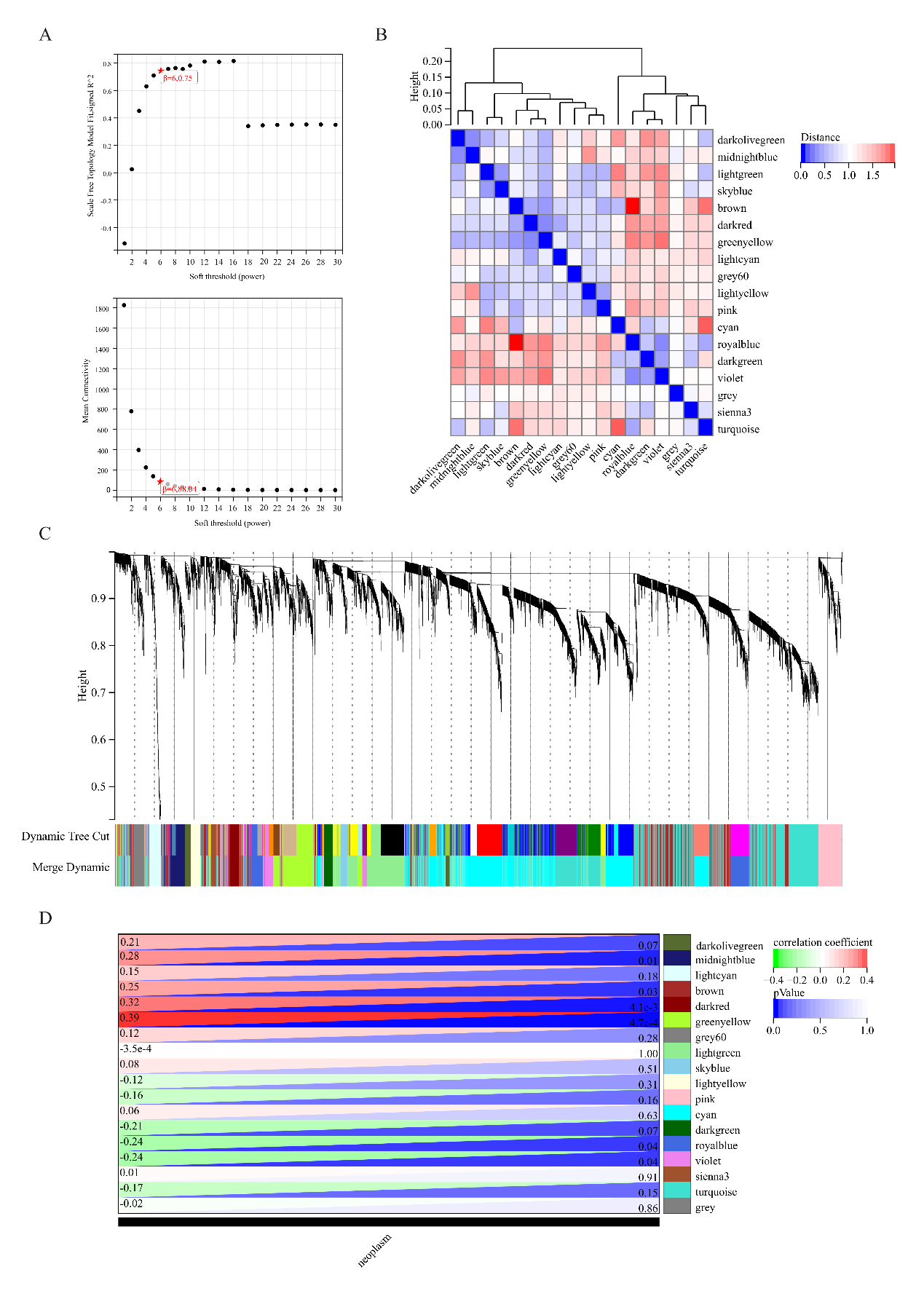


**Supplementary Figure 4.** WGCNA analysis. (A) β = 6,0.75. β = 6,88.04; (B, C) The hierarchical clustering tree of all genes was constructed, and 18 important modules were generated; (D) The heat map of correlation between modules and phenotypes.


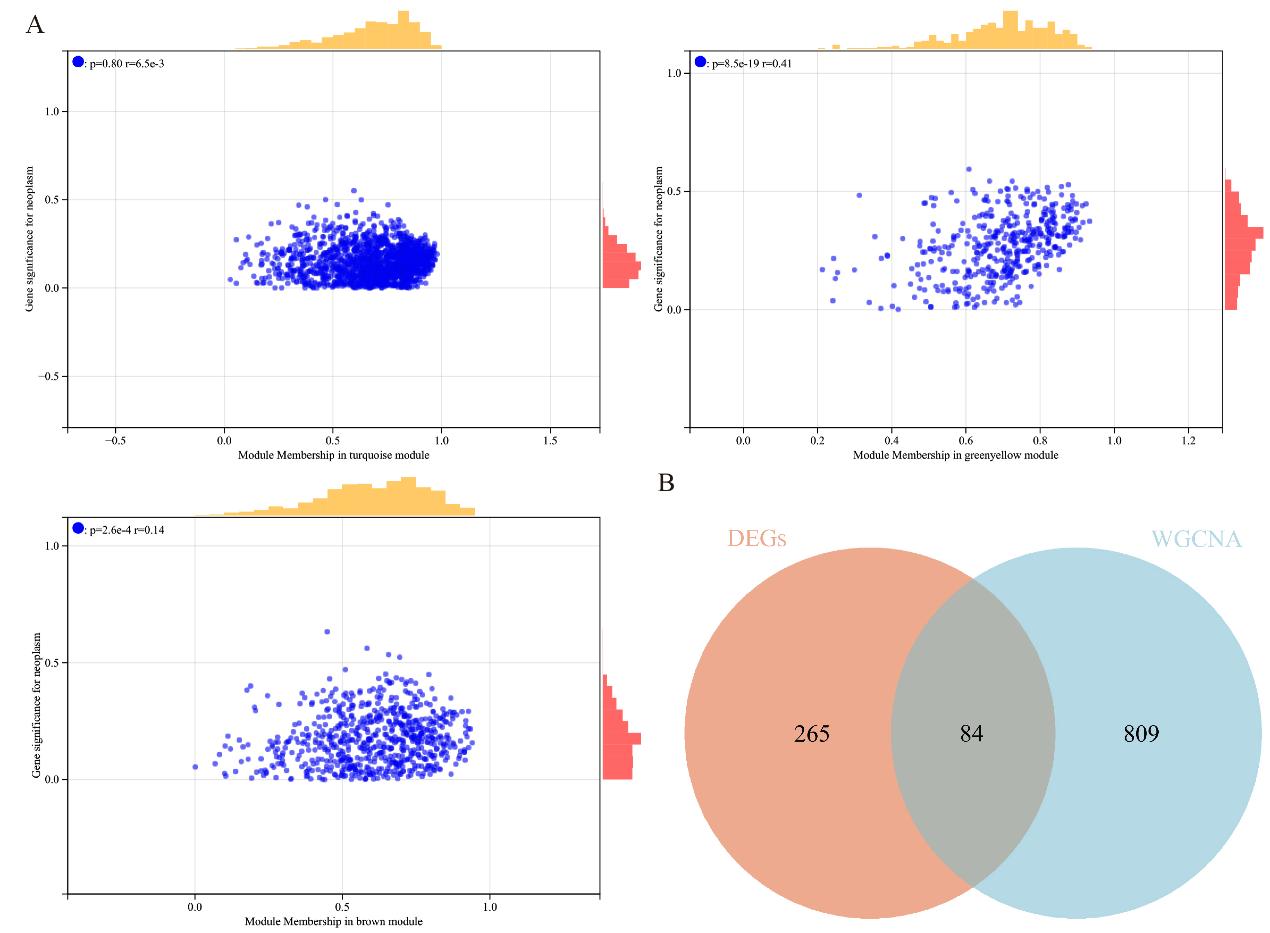


**Supplementary Figure 5.** (A) The scatter map of correlation between gene significance and module membership of related hub genes. (B) The DEGs screened by WGCNA and DEGs was used to obtain venn map 84 intersection genes were obtained.


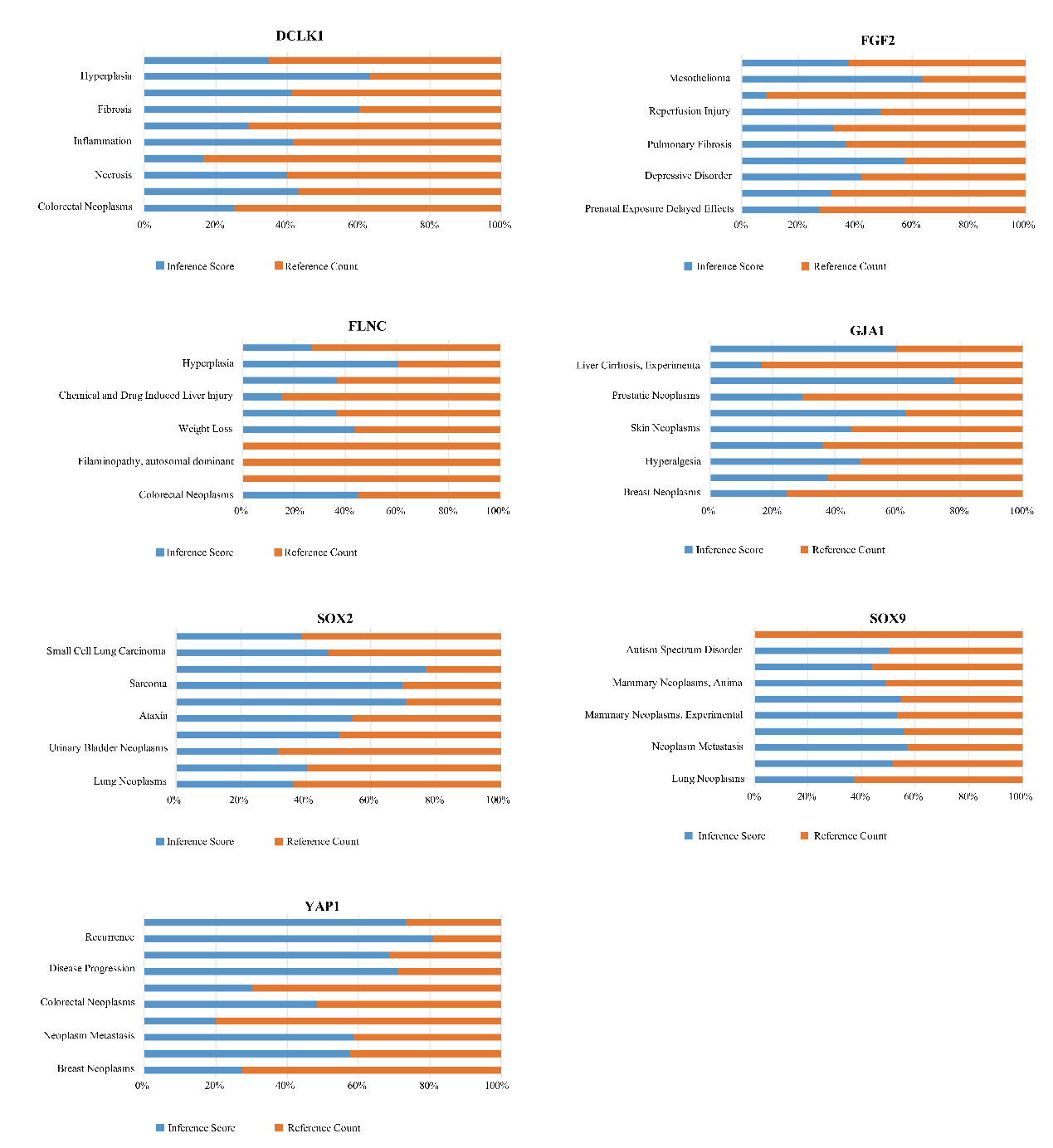


**Supplementary Figure 6.** Comparative toxicogenomics database analysis.


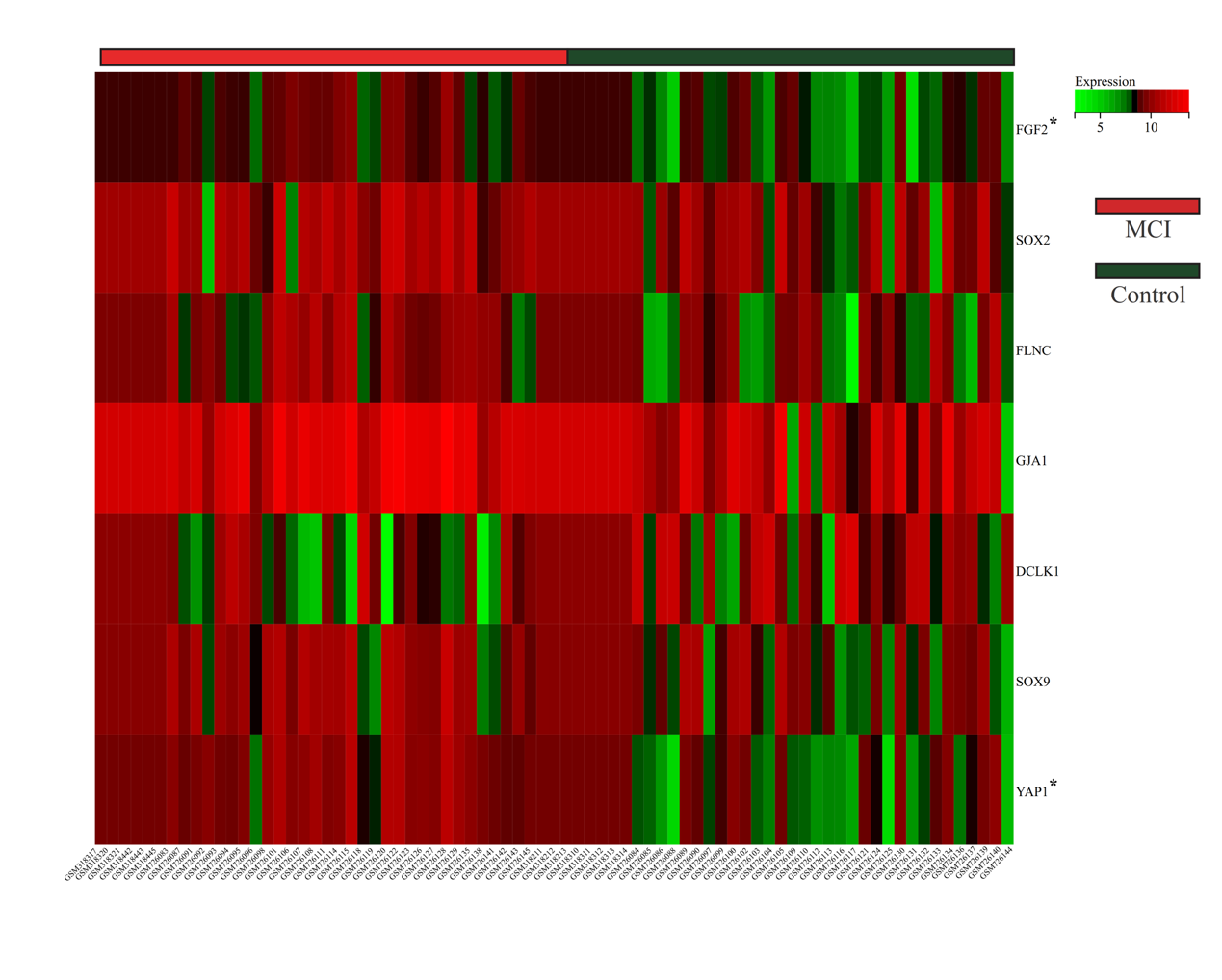


**Supplementary Figure 7.** The heatmap depicting the expression levels of differentially expressed genes related to ferroptosis in the merged matrix of GSE29378 and GSE12685 datasets. YAP1 (FDR = 0.003), FGF2 (FDR = 0.017), SOX2 (FDR = 0.217), FLNC (FDR = 0.184), GJA1 (FDR = 0.085), DCLK1 (FDR = 0.085), SOX9 (FDR = 0.264). * indicates statistical significance with *P* < 0.05. FDR, false discovery rate.

## 1.2 Supplementary Tables

**Supplementary Table. The raw data table of individual MoCA domain scores for the enrolled population**

| **MCI** | **FGF2** | **YAP1** | **MoCA** | **Visuospatial Executive** | **Naming** | **Attention** | **Language** | **Abstraction** | **Delayed Recall** | **Orientation** |
| --- | --- | --- | --- | --- | --- | --- | --- | --- | --- | --- |
| .00 | 7.93 | 58.49 | 27.00 | 4.00 | 3.00 | 6.00 | 2.00 | 1.00 | 4.00 | 6.00 |
| .00 | 6.33 | 86.99 | 27.00 | 4.00 | 3.00 | 6.00 | 2.00 | 1.00 | 4.00 | 6.00 |
| .00 | 5.85 | 57.61 | 28.00 | 5.00 | 1.00 | 6.00 | 3.00 | 2.00 | 4.00 | 6.00 |
| .00 | 4.59 | 144.09 | 27.00 | 4.00 | 3.00 | 6.00 | 2.00 | 2.00 | 3.00 | 6.00 |
| .00 | 6.65 | 49.68 | 27.00 | 4.00 | 3.00 | 6.00 | 3.00 | 1.00 | 3.00 | 6.00 |
| .00 | 7.04 | 73.00 | 29.00 | 5.00 | 3.00 | 6.00 | 2.00 | 1.00 | 5.00 | 6.00 |
| .00 | 10.38 | 177.71 | 28.00 | 4.00 | 3.00 | 6.00 | 2.00 | 1.00 | 5.00 | 6.00 |
| .00 | 6.65 | 82.55 | 29.00 | 5.00 | 3.00 | 6.00 | 2.00 | 2.00 | 4.00 | 6.00 |
| .00 | 9.86 | 86.26 | 28.00 | 4.00 | 3.00 | 6.00 | 2.00 | 2.00 | 4.00 | 6.00 |
| .00 | 11.04 | 76.05 | 28.00 | 5.00 | 3.00 | 6.00 | 1.00 | 1.00 | 5.00 | 6.00 |
| .00 | 6.17 | 70.46 | 29.00 | 5.00 | 3.00 | 5.00 | 3.00 | 2.00 | 4.00 | 6.00 |
| .00 | 7.61 | 89.88 | 27.00 | 4.00 | 2.00 | 6.00 | 3.00 | 1.00 | 4.00 | 6.00 |
| .00 | 5.53 | 74.52 | 27.00 | 4.00 | 3.00 | 6.00 | 2.00 | 2.00 | 3.00 | 6.00 |
| .00 | 6.42 | 87.56 | 29.00 | 5.00 | 2.00 | 6.00 | 3.00 | 2.00 | 4.00 | 6.00 |
| .00 | 8.77 | 87.99 | 27.00 | 4.00 | 2.00 | 6.00 | 3.00 | 2.00 | 3.00 | 6.00 |
| .00 | 7.13 | 79.37 | 28.00 | 5.00 | 3.00 | 6.00 | 3.00 | 2.00 | 2.00 | 6.00 |
| .00 | 11.37 | 72.20 | 27.00 | 5.00 | 3.00 | 6.00 | 2.00 | .00 | 4.00 | 6.00 |
| .00 | 7.61 | 83.77 | 28.00 | 3.00 | 2.00 | 6.00 | 3.00 | 2.00 | 5.00 | 6.00 |
| .00 | 13.54 | 63.16 | 28.00 | 5.00 | 1.00 | 6.00 | 3.00 | 2.00 | 4.00 | 6.00 |
| .00 | 8.42 | 78.09 | 28.00 | 5.00 | 3.00 | 5.00 | 2.00 | 2.00 | 4.00 | 6.00 |
| .00 | 8.26 | 57.17 | 28.00 | 5.00 | 3.00 | 6.00 | 2.00 | 2.00 | 3.00 | 6.00 |
| .00 | 6.33 | 97.99 | 27.00 | 5.00 | 3.00 | 6.00 | 2.00 | 2.00 | 3.00 | 6.00 |
| .00 | 6.81 | 94.93 | 28.00 | 5.00 | 3.00 | 6.00 | 3.00 | .00 | 4.00 | 6.00 |
| .00 | 14.89 | 70.61 | 29.00 | 5.00 | 3.00 | 6.00 | 3.00 | 2.00 | 4.00 | 5.00 |
| .00 | 6.65 | 72.98 | 30.00 | 5.00 | 3.00 | 6.00 | 2.00 | 2.00 | 5.00 | 6.00 |
| .00 | 7.13 | 102.05 | 28.00 | 5.00 | 3.00 | 6.00 | 2.00 | 2.00 | 3.00 | 6.00 |
| .00 | 6.65 | 77.72 | 27.00 | 3.00 | 3.00 | 6.00 | 2.00 | 2.00 | 4.00 | 6.00 |
| .00 | 10.22 | 84.42 | 28.00 | 5.00 | 3.00 | 6.00 | 2.00 | 1.00 | 4.00 | 6.00 |
| .00 | 7.93 | 72.82 | 27.00 | 5.00 | 3.00 | 5.00 | 1.00 | 2.00 | 5.00 | 6.00 |
| .00 | 6.81 | 93.43 | 28.00 | 2.00 | 3.00 | 6.00 | 3.00 | 2.00 | 5.00 | 6.00 |
| .00 | 6.90 | 82.24 | 29.00 | 5.00 | 3.00 | 6.00 | 2.00 | 2.00 | 5.00 | 6.00 |
| .00 | 11.42 | 66.56 | 28.00 | 4.00 | 3.00 | 6.00 | 2.00 | 2.00 | 5.00 | 6.00 |
| .00 | 6.65 | 63.33 | 27.00 | 5.00 | 3.00 | 6.00 | 3.00 | 2.00 | 3.00 | 5.00 |
| .00 | 7.13 | 66.57 | 27.00 | 5.00 | 3.00 | 6.00 | 2.00 | 2.00 | 3.00 | 6.00 |
| .00 | 6.17 | 65.10 | 27.00 | 5.00 | 3.00 | 6.00 | 2.00 | 2.00 | 2.00 | 6.00 |
| .00 | 4.43 | 54.66 | 29.00 | 5.00 | 3.00 | 5.00 | 3.00 | 1.00 | 5.00 | 6.00 |
| .00 | 4.12 | 88.53 | 29.00 | 5.00 | 3.00 | 6.00 | 3.00 | 2.00 | 3.00 | 6.00 |
| .00 | 2.88 | 81.93 | 28.00 | 4.00 | 3.00 | 6.00 | 2.00 | 2.00 | 5.00 | 6.00 |
| .00 | 7.13 | 113.46 | 27.00 | 5.00 | 3.00 | 6.00 | 2.00 | 2.00 | 3.00 | 6.00 |
| .00 | 7.29 | 112.18 | 27.00 | 5.00 | 3.00 | 5.00 | 3.00 | 3.00 | 3.00 | 5.00 |
| .00 | 6.16 | 91.00 | 27.00 | 5.00 | 3.00 | 5.00 | 3.00 | 2.00 | 2.00 | 6.00 |
| .00 | 15.40 | 103.16 | 30.00 | 5.00 | 3.00 | 6.00 | 3.00 | 2.00 | 4.00 | 6.00 |
| .00 | 5.06 | 57.03 | 27.00 | 4.00 | 3.00 | 6.00 | 2.00 | 2.00 | 3.00 | 6.00 |
| .00 | 5.85 | 62.45 | 29.00 | 5.00 | 3.00 | 6.00 | 3.00 | 2.00 | 3.00 | 6.00 |
| .00 | 3.04 | 81.93 | 27.00 | 5.00 | 3.00 | 5.00 | 2.00 | 2.00 | 4.00 | 6.00 |
| .00 | 3.50 | 130.35 | 27.00 | 5.00 | 3.00 | 6.00 | 1.00 | 2.00 | 4.00 | 6.00 |
| .00 | 4.12 | 56.89 | 27.00 | 5.00 | 3.00 | 6.00 | 3.00 | 2.00 | 1.00 | 6.00 |
| .00 | 9.23 | 109.51 | 27.00 | 4.00 | 3.00 | 6.00 | 2.00 | 2.00 | 3.00 | 6.00 |
| .00 | 6.65 | 90.84 | 28.00 | 5.00 | 3.00 | 6.00 | 2.00 | 2.00 | 4.00 | 6.00 |
| .00 | 6.49 | 81.63 | 26.00 | 5.00 | 3.00 | 6.00 | 3.00 | 2.00 | 1.00 | 6.00 |
| .00 | 5.06 | 103.91 | 28.00 | 5.00 | 3.00 | 6.00 | 2.00 | 2.00 | 4.00 | 6.00 |
| .00 | 3.65 | 83.46 | 27.00 | 4.00 | 3.00 | 5.00 | 2.00 | 2.00 | 4.00 | 6.00 |
| .00 | 5.53 | 67.48 | 28.00 | 4.00 | 3.00 | 6.00 | 3.00 | 1.00 | 4.00 | 6.00 |
| .00 | 4.28 | 93.17 | 28.00 | 5.00 | 3.00 | 6.00 | 3.00 | 2.00 | 2.00 | 6.00 |
| 1.00 | 31.66 | 417.42 | 25.00 | 5.00 | 3.00 | 5.00 | 3.00 | 2.00 | 1.00 | 6.00 |
| 1.00 | 24.01 | 404.43 | 20.00 | 2.00 | 2.00 | 5.00 | 1.00 | .00 | 3.00 | 6.00 |
| 1.00 | 32.02 | 940.71 | 23.00 | 4.00 | 3.00 | 4.00 | 1.00 | 2.00 | 2.00 | 6.00 |
| 1.00 | 18.96 | 603.67 | 25.00 | 5.00 | 2.00 | 6.00 | 2.00 | 2.00 | 1.00 | 6.00 |
| 1.00 | 38.79 | 80.57 | 22.00 | 3.00 | 3.00 | 6.00 | 1.00 | 2.00 | 1.00 | 6.00 |
| 1.00 | 6.59 | 534.50 | 20.00 | 4.00 | 3.00 | 4.00 | 1.00 | 2.00 | .00 | 6.00 |
| 1.00 | 22.60 | 144.96 | 25.00 | 2.00 | 2.00 | 6.00 | 2.00 | 1.00 | 5.00 | 6.00 |
| 1.00 | 24.89 | 133.35 | 23.00 | 3.00 | 3.00 | 6.00 | 2.00 | 1.00 | 1.00 | 6.00 |
| 1.00 | 21.56 | 268.75 | 22.00 | 5.00 | 3.00 | 5.00 | 1.00 | 2.00 | .00 | 6.00 |
| 1.00 | 18.62 | 187.38 | 21.00 | 4.00 | 3.00 | 4.00 | 1.00 | 2.00 | .00 | 6.00 |
| 1.00 | 20.00 | 104.96 | 25.00 | 3.00 | 3.00 | 6.00 | 3.00 | 2.00 | 1.00 | 6.00 |
| 1.00 | 16.05 | 344.17 | 22.00 | 5.00 | 3.00 | 6.00 | 1.00 | 1.00 | .00 | 6.00 |
| 1.00 | 13.69 | 359.70 | 20.00 | 4.00 | 3.00 | 6.00 | 1.00 | .00 | .00 | 5.00 |
| 1.00 | 12.85 | 219.91 | 25.00 | 4.00 | 3.00 | 6.00 | 2.00 | 1.00 | 2.00 | 6.00 |
| 1.00 | 19.83 | 504.96 | 24.00 | 3.00 | 3.00 | 4.00 | 2.00 | 2.00 | 4.00 | 6.00 |
| 1.00 | 12.35 | 641.68 | 23.00 | 4.00 | 3.00 | 5.00 | 2.00 | 2.00 | 1.00 | 5.00 |
| 1.00 | 12.18 | 1191.88 | 24.00 | 4.00 | 3.00 | 6.00 | 1.00 | 1.00 | 2.00 | 6.00 |
| 1.00 | 12.35 | 1933.53 | 24.00 | 4.00 | 2.00 | 6.00 | 2.00 | 2.00 | 1.00 | 6.00 |
| 1.00 | 12.18 | 94.03 | 25.00 | 4.00 | 3.00 | 5.00 | 2.00 | 1.00 | 3.00 | 6.00 |
| 1.00 | 13.02 | 140.05 | 18.00 | 2.00 | 3.00 | 5.00 | 2.00 | .00 | .00 | 5.00 |
| 1.00 | 12.85 | 262.84 | 24.00 | 4.00 | 3.00 | 6.00 | 2.00 | 1.00 | 2.00 | 6.00 |
| 1.00 | 8.71 | 132.35 | 23.00 | 3.00 | 3.00 | 5.00 | 2.00 | 1.00 | 2.00 | 6.00 |
| 1.00 | 21.91 | 510.56 | 23.00 | 5.00 | 3.00 | 5.00 | 1.00 | 2.00 | .00 | 6.00 |
| 1.00 | 19.48 | 104.96 | 25.00 | 3.00 | 2.00 | 6.00 | 2.00 | 2.00 | 4.00 | 6.00 |
| 1.00 | 17.42 | 161.19 | 24.00 | 5.00 | 2.00 | 5.00 | 2.00 | 2.00 | 2.00 | 6.00 |
| 1.00 | 16.05 | 159.27 | 24.00 | 4.00 | 3.00 | 6.00 | 1.00 | .00 | 4.00 | 5.00 |
| 1.00 | 14.53 | 118.70 | 25.00 | 5.00 | 2.00 | 6.00 | 2.00 | 2.00 | 1.00 | 6.00 |
| 1.00 | 14.87 | 140.79 | 25.00 | 5.00 | 3.00 | 6.00 | 3.00 | 2.00 | .00 | 6.00 |
| 1.00 | 15.38 | 133.85 | 25.00 | 5.00 | 2.00 | 6.00 | 2.00 | 2.00 | 2.00 | 5.00 |
| 1.00 | 17.76 | 264.81 | 24.00 | 3.00 | 3.00 | 6.00 | 2.00 | 2.00 | 1.00 | 6.00 |
| 1.00 | 19.48 | 197.33 | 20.00 | 4.00 | 3.00 | 5.00 | 1.00 | 2.00 | .00 | 5.00 |
| 1.00 | 17.42 | 615.06 | 23.00 | 3.00 | 2.00 | 5.00 | 3.00 | 2.00 | 2.00 | 6.00 |
| 1.00 | 22.08 | 144.47 | 24.00 | 5.00 | 3.00 | 6.00 | 1.00 | 2.00 | 1.00 | 6.00 |
| 1.00 | 21.56 | 131.60 | 21.00 | 3.00 | 2.00 | 6.00 | 2.00 | 1.00 | .00 | 6.00 |
| 1.00 | 19.14 | 112.78 | 21.00 | 3.00 | 3.00 | 6.00 | 1.00 | 1.00 | .00 | 6.00 |
| 1.00 | 12.51 | 272.69 | 21.00 | 5.00 | 1.00 | 4.00 | 3.00 | 1.00 | .00 | 6.00 |
| 1.00 | 18.28 | 148.14 | 24.00 | 4.00 | 2.00 | 5.00 | 1.00 | 1.00 | 4.00 | 6.00 |
| 1.00 | 9.69 | 451.73 | 25.00 | 4.00 | 2.00 | 6.00 | 2.00 | 2.00 | 2.00 | 6.00 |
| 1.00 | 9.03 | 391.87 | 19.00 | 3.00 | 2.00 | 4.00 | 1.00 | 1.00 | 1.00 | 6.00 |
| 1.00 | 15.88 | 222.17 | 21.00 | 4.00 | 3.00 | 4.00 | 1.00 | 2.00 | 1.00 | 6.00 |
| 1.00 | 11.18 | 385.02 | 25.00 | 4.00 | 3.00 | 5.00 | 2.00 | 2.00 | 3.00 | 6.00 |
| 1.00 | 14.70 | 145.70 | 25.00 | 4.00 | 3.00 | 6.00 | 2.00 | 1.00 | 2.00 | 6.00 |
| 1.00 | 17.93 | 116.91 | 25.00 | 4.00 | 3.00 | 6.00 | 3.00 | .00 | 3.00 | 6.00 |
| 1.00 | 20.34 | 268.54 | 21.00 | 4.00 | 1.00 | 4.00 | 3.00 | 1.00 | 1.00 | 6.00 |
| 1.00 | 19.65 | 779.55 | 24.00 | 4.00 | 2.00 | 6.00 | 3.00 | 2.00 | .00 | 6.00 |
| 1.00 | 13.52 | 137.33 | 23.00 | 5.00 | 2.00 | 6.00 | 2.00 | 2.00 | .00 | 6.00 |
| 1.00 | 15.55 | 161.91 | 24.00 | 5.00 | 1.00 | 6.00 | 1.00 | 1.00 | 3.00 | 6.00 |
| 1.00 | 12.68 | 137.33 | 24.00 | 3.00 | 3.00 | 4.00 | 3.00 | 1.00 | 4.00 | 6.00 |
| 1.00 | 9.36 | 280.96 | 22.00 | 2.00 | 3.00 | 6.00 | 1.00 | 1.00 | 2.00 | 6.00 |
| 1.00 | 8.54 | 169.53 | 20.00 | 3.00 | 3.00 | 5.00 | 1.00 | .00 | 2.00 | 5.00 |
| 1.00 | 19.31 | 167.63 | 25.00 | 4.00 | 3.00 | 6.00 | 2.00 | 2.00 | 2.00 | 6.00 |
| 1.00 | 30.22 | 481.86 | 24.00 | 3.00 | 2.00 | 6.00 | 2.00 | 1.00 | 3.00 | 6.00 |
| 1.00 | 18.45 | 293.30 | 22.00 | 5.00 | 1.00 | 6.00 | 2.00 | 1.00 | .00 | 6.00 |
| 1.00 | 10.68 | 453.72 | 21.00 | 4.00 | 2.00 | 5.00 | 2.00 | 1.00 | .00 | 6.00 |
| 1.00 | 23.30 | 144.23 | 24.00 | 5.00 | 3.00 | 6.00 | .00 | 1.00 | 2.00 | 6.00 |
| 1.00 | 18.10 | 138.56 | 24.00 | 4.00 | 3.00 | 6.00 | 2.00 | 2.00 | .00 | 6.00 |
| 1.00 | 14.02 | 123.79 | 25.00 | 3.00 | 3.00 | 5.00 | 1.00 | 2.00 | 4.00 | 6.00 |
| 1.00 | 12.18 | 114.33 | 25.00 | 5.00 | 1.00 | 5.00 | 2.00 | 2.00 | 3.00 | 6.00 |
| 1.00 | 24.53 | 766.24 | 23.00 | 3.00 | 3.00 | 6.00 | 2.00 | 1.00 | 1.00 | 6.00 |
| 1.00 | 9.69 | 520.06 | 23.00 | 2.00 | 3.00 | 5.00 | 2.00 | 2.00 | 2.00 | 6.00 |
| 1.00 | 13.02 | 123.79 | 23.00 | 5.00 | 3.00 | 5.00 | 3.00 | 1.00 | .00 | 6.00 |
| 1.00 | 26.65 | 282.52 | 23.00 | 4.00 | 1.00 | 6.00 | 1.00 | 1.00 | 3.00 | 6.00 |
| 1.00 | 15.55 | 251.39 | 24.00 | 4.00 | 3.00 | 5.00 | 3.00 | 1.00 | 1.00 | 6.00 |
| 1.00 | 16.05 | 493.49 | 25.00 | 4.00 | 3.00 | 6.00 | 2.00 | .00 | 3.00 | 6.00 |
| 1.00 | 10.52 | 328.09 | 18.00 | 3.00 | .00 | 6.00 | 2.00 | .00 | .00 | 6.00 |
| 1.00 | 14.02 | 398.86 | 20.00 | 2.00 | 3.00 | 5.00 | 1.00 | 1.00 | 2.00 | 5.00 |
| 1.00 | 14.36 | 322.53 | 19.00 | 2.00 | 1.00 | 6.00 | 2.00 | 1.00 | .00 | 6.00 |
| 1.00 | 13.52 | 527.07 | 24.00 | 5.00 | 3.00 | 6.00 | 3.00 | .00 | .00 | 6.00 |
| 1.00 | 15.04 | 256.69 | 21.00 | 4.00 | 2.00 | 5.00 | 1.00 | 2.00 | 1.00 | 6.00 |
| 1.00 | 14.02 | 193.87 | 19.00 | 3.00 | 3.00 | 5.00 | 2.00 | .00 | .00 | 5.00 |
| 1.00 | 15.21 | 270.28 | 22.00 | 5.00 | 3.00 | 6.00 | 2.00 | 1.00 | 1.00 | 3.00 |
| 1.00 | 12.51 | 434.07 | 25.00 | 4.00 | 1.00 | 6.00 | 2.00 | 2.00 | 3.00 | 6.00 |
| 1.00 | 12.85 | 357.04 | 22.00 | 2.00 | 3.00 | 6.00 | 3.00 | 1.00 | .00 | 6.00 |
| 1.00 | 17.42 | 284.00 | 18.00 | 2.00 | 2.00 | 4.00 | 1.00 | .00 | 2.00 | 6.00 |
| 1.00 | 15.04 | 116.91 | 21.00 | 3.00 | 3.00 | 4.00 | 1.00 | 2.00 | 1.00 | 6.00 |
| 1.00 | 10.85 | 675.41 | 25.00 | 5.00 | 2.00 | 6.00 | 2.00 | 1.00 | 2.00 | 6.00 |
| 1.00 | 12.01 | 506.54 | 22.00 | 5.00 | 3.00 | 4.00 | 2.00 | .00 | 1.00 | 6.00 |
| 1.00 | 17.59 | 328.03 | 23.00 | 4.00 | 3.00 | 5.00 | 1.00 | 2.00 | 2.00 | 5.00 |
| 1.00 | 13.86 | 296.72 | 20.00 | 4.00 | 3.00 | 5.00 | 1.00 | 1.00 | .00 | 6.00 |
| 1.00 | 11.68 | 200.78 | 22.00 | 4.00 | 3.00 | 6.00 | 2.00 | .00 | .00 | 6.00 |
| 1.00 | 16.40 | 148.87 | 24.00 | 5.00 | 3.00 | 6.00 | 1.00 | 2.00 | 1.00 | 6.00 |
| 1.00 | 12.77 | 361.92 | 24.00 | 5.00 | 3.00 | 5.00 | 2.00 | 2.00 | 1.00 | 6.00 |
| 1.00 | 24.21 | 359.95 | 24.00 | 4.00 | 2.00 | 6.00 | 3.00 | 1.00 | 2.00 | 6.00 |
| 1.00 | 15.46 | 382.49 | 21.00 | 4.00 | 3.00 | 6.00 | 1.00 | .00 | .00 | 6.00 |
| 1.00 | 10.80 | 379.80 | 24.00 | 3.00 | 3.00 | 6.00 | .00 | 2.00 | 4.00 | 6.00 |
| 1.00 | 9.92 | 219.21 | 22.00 | 4.00 | 3.00 | 5.00 | 1.00 | 2.00 | 1.00 | 5.00 |
| 1.00 | 9.92 | 108.63 | 18.00 | 4.00 | 1.00 | 4.00 | 2.00 | .00 | .00 | 6.00 |
| 1.00 | 10.45 | 99.22 | 21.00 | 4.00 | 1.00 | 5.00 | 1.00 | 1.00 | 2.00 | 6.00 |
| 1.00 | 16.00 | 370.68 | 21.00 | 3.00 | 3.00 | 5.00 | 2.00 | 1.00 | .00 | 6.00 |
| 1.00 | 20.18 | 780.01 | 25.00 | 4.00 | 2.00 | 6.00 | 2.00 | .00 | 4.00 | 6.00 |
| 1.00 | 11.87 | 281.23 | 20.00 | 3.00 | 3.00 | 5.00 | 2.00 | 1.00 | .00 | 6.00 |
| 1.00 | 7.61 | 290.70 | 20.00 | 4.00 | 1.00 | 1.00 | 1.00 | 1.00 | 5.00 | 6.00 |
| 1.00 | 5.50 | 215.97 | 24.00 | 3.00 | 1.00 | 5.00 | 1.00 | 2.00 | 5.00 | 6.00 |
| 1.00 | 7.97 | 266.23 | 19.00 | 2.00 | 2.00 | 4.00 | 3.00 | 1.00 | .00 | 6.00 |
| 1.00 | 8.14 | 248.98 | 22.00 | 1.00 | 3.00 | 6.00 | 3.00 | 2.00 | .00 | 6.00 |
| 1.00 | 5.68 | 100.36 | 22.00 | 3.00 | 3.00 | 6.00 | 2.00 | 1.00 | .00 | 6.00 |
| 1.00 | 10.09 | 215.97 | 23.00 | 4.00 | 3.00 | 6.00 | 3.00 | .00 | .00 | 6.00 |
| 1.00 | 7.08 | 288.42 | 25.00 | 5.00 | 3.00 | 5.00 | 2.00 | 2.00 | 2.00 | 5.00 |
| 1.00 | 16.00 | 112.93 | 19.00 | 3.00 | 1.00 | 3.00 | 2.00 | 2.00 | 1.00 | 6.00 |
| 1.00 | 9.74 | 161.72 | 18.00 | 3.00 | 1.00 | 5.00 | 1.00 | .00 | 1.00 | 6.00 |
| 1.00 | 6.91 | 2693.65 | 22.00 | 3.00 | 3.00 | 5.00 | 2.00 | 2.00 | .00 | 6.00 |
| 1.00 | 10.09 | 269.74 | 21.00 | 4.00 | 3.00 | 4.00 | 2.00 | 1.00 | .00 | 6.00 |
| 1.00 | 12.77 | 179.61 | 23.00 | 4.00 | 3.00 | 5.00 | 3.00 | 1.00 | .00 | 6.00 |
| 1.00 | 11.52 | 195.58 | 18.00 | 2.00 | 2.00 | 5.00 | 1.00 | 1.00 | .00 | 6.00 |
| 1.00 | 14.92 | 195.22 | 23.00 | 4.00 | 3.00 | 5.00 | 2.00 | 1.00 | 1.00 | 6.00 |
| 1.00 | 12.41 | 159.07 | 22.00 | 3.00 | 3.00 | 6.00 | 2.00 | 2.00 | .00 | 6.00 |
| 1.00 | 8.67 | 65.84 | 25.00 | 4.00 | 3.00 | 5.00 | 2.00 | 2.00 | 3.00 | 6.00 |
| 1.00 | 18.36 | 153.40 | 22.00 | 3.00 | 3.00 | 6.00 | 3.00 | .00 | .00 | 6.00 |
| 1.00 | 8.67 | 889.19 | 25.00 | 5.00 | 3.00 | 6.00 | 3.00 | 2.00 | .00 | 6.00 |
| 1.00 | 7.97 | 498.27 | 23.00 | 3.00 | 3.00 | 4.00 | 2.00 | 2.00 | 2.00 | 6.00 |
| 1.00 | 16.73 | 116.29 | 23.00 | 3.00 | 2.00 | 6.00 | 2.00 | .00 | 3.00 | 6.00 |
| 1.00 | 7.97 | 261.33 | 25.00 | 3.00 | 3.00 | 6.00 | 2.00 | 2.00 | 3.00 | 6.00 |
| 1.00 | 10.98 | 383.27 | 21.00 | 4.00 | 3.00 | 4.00 | 2.00 | 1.00 | .00 | 6.00 |
| 1.00 | 15.46 | 194.88 | 24.00 | 4.00 | 3.00 | 6.00 | 2.00 | .00 | 2.00 | 6.00 |
| 1.00 | 10.09 | 185.93 | 25.00 | 5.00 | 3.00 | 6.00 | 1.00 | 1.00 | 2.00 | 6.00 |
| 1.00 | 9.21 | 145.51 | 23.00 | 5.00 | 3.00 | 5.00 | 2.00 | 2.00 | .00 | 5.00 |
| 1.00 | 13.31 | 664.30 | 24.00 | 3.00 | 3.00 | 5.00 | 1.00 | 2.00 | 4.00 | 6.00 |
| 1.00 | 16.73 | 214.17 | 23.00 | 3.00 | 3.00 | 6.00 | 2.00 | .00 | 2.00 | 6.00 |
| 1.00 | 11.16 | 108.07 | 25.00 | 4.00 | 3.00 | 5.00 | 1.00 | 2.00 | 3.00 | 6.00 |
| 1.00 | 12.23 | 285.74 | 21.00 | 3.00 | 3.00 | 5.00 | 1.00 | 1.00 | 2.00 | 5.00 |
| 1.00 | 10.09 | 167.21 | 25.00 | 5.00 | 3.00 | 6.00 | 2.00 | 2.00 | .00 | 6.00 |
| 1.00 | 33.73 | 273.05 | 24.00 | 4.00 | 3.00 | 6.00 | 2.00 | 2.00 | 1.00 | 6.00 |
| 1.00 | 7.26 | 195.06 | 25.00 | 5.00 | 3.00 | 5.00 | 2.00 | 2.00 | 3.00 | 5.00 |
| 1.00 | 6.73 | 194.88 | 24.00 | 4.00 | 3.00 | 6.00 | .00 | 2.00 | 2.00 | 6.00 |
| 1.00 | 9.03 | 280.57 | 24.00 | 5.00 | 3.00 | 6.00 | 2.00 | 1.00 | .00 | 6.00 |
| 1.00 | 10.80 | 261.72 | 21.00 | 4.00 | 3.00 | 4.00 | 1.00 | 2.00 | .00 | 6.00 |
| 1.00 | 10.98 | 142.76 | 24.00 | 5.00 | 3.00 | 6.00 | 2.00 | 2.00 | .00 | 6.00 |
| 1.00 | 9.74 | 231.27 | 22.00 | 4.00 | 3.00 | 6.00 | 1.00 | 1.00 | .00 | 6.00 |
| 1.00 | 24.76 | 186.53 | 22.00 | 2.00 | 3.00 | 5.00 | 1.00 | 2.00 | 2.00 | 6.00 |
| 1.00 | 19.99 | 233.61 | 25.00 | 5.00 | 3.00 | 6.00 | 3.00 | 1.00 | 1.00 | 6.00 |
| 1.00 | 10.45 | 264.67 | 23.00 | 4.00 | 3.00 | 5.00 | 3.00 | 1.00 | 1.00 | 5.00 |
| 1.00 | 14.38 | 857.87 | 23.00 | 3.00 | 3.00 | 6.00 | 1.00 | 2.00 | 2.00 | 6.00 |
| 1.00 | 11.16 | 124.11 | 24.00 | 2.00 | 3.00 | 4.00 | 3.00 | 2.00 | 3.00 | 6.00 |
| 1.00 | 7.26 | 122.06 | 23.00 | 2.00 | 3.00 | 5.00 | 2.00 | 2.00 | 3.00 | 6.00 |
| 1.00 | 15.28 | 107.69 | 25.00 | 4.00 | 2.00 | 6.00 | 2.00 | .00 | 4.00 | 6.00 |
| 1.00 | 7.79 | 166.62 | 23.00 | 4.00 | 2.00 | 6.00 | 1.00 | .00 | 3.00 | 6.00 |
| 1.00 | 16.00 | 784.09 | 20.00 | 4.00 | 2.00 | 5.00 | 2.00 | 1.00 | .00 | 6.00 |
| 1.00 | 26.80 | 417.37 | 24.00 | 3.00 | 2.00 | 5.00 | 3.00 | 2.00 | 2.00 | 6.00 |
| 1.00 | 8.32 | 166.43 | 21.00 | 4.00 | 2.00 | 4.00 | 2.00 | 2.00 | .00 | 6.00 |
| 1.00 | 8.85 | 117.22 | 22.00 | 4.00 | 3.00 | 6.00 | 1.00 | 2.00 | .00 | 5.00 |
| 1.00 | 8.32 | 101.11 | 21.00 | 3.00 | .00 | 6.00 | 3.00 | 2.00 | 1.00 | 6.00 |
| 1.00 | 8.14 | 184.41 | 19.00 | 3.00 | 2.00 | 5.00 | 1.00 | 1.00 | 1.00 | 6.00 |
| 1.00 | 7.08 | 187.27 | 18.00 | 2.00 | 3.00 | 4.00 | 1.00 | 1.00 | .00 | 6.00 |
| 1.00 | 9.92 | 140.00 | 18.00 | 3.00 | 2.00 | 4.00 | 1.00 | 1.00 | .00 | 6.00 |
| 1.00 | 8.14 | 139.00 | 24.00 | 4.00 | 3.00 | 4.00 | 1.00 | 1.00 | 4.00 | 6.00 |
| 1.00 | 13.31 | 143.51 | 24.00 | 4.00 | 3.00 | 5.00 | 2.00 | 1.00 | 2.00 | 6.00 |

MCI, Mild cognitive impairment; MoCA, Montreal Cognitive Assessment; In the "MCI" column, 0 indicates the control group, and 1 indicates the MCI group.
